# Neurocomputational mechanisms at play when weighing concerns for extrinsic rewards, moral values, and social image

**DOI:** 10.1371/journal.pbio.3000283

**Published:** 2019-06-06

**Authors:** Chen Qu, Elise Météreau, Luigi Butera, Marie Claire Villeval, Jean-Claude Dreher

**Affiliations:** 1 Center for Studies of Psychological Application, School of Psychology, South China Normal University, Guangzhou, China; 2 Institut des Sciences Cognitives Marc Jeannerod, ‘Neuroeconomics, *Reward and Decision Making Group*,*’* Centre National de la Recherche Scientifique, UMR 5229, Bron, France; 3 Université Claude Bernard Lyon 1, Lyon, France; 4 University of Lyon, CNRS, GATE (UMR 5824), Ecully, France; 5 IZA, Bonn, Germany; Oxford University, UNITED KINGDOM

## Abstract

Humans not only value extrinsic monetary rewards but also their own morality and their image in the eyes of others. Yet violating moral norms is frequent, especially when people know that they are not under scrutiny. When moral values and monetary payoffs are at odds, how does the brain weigh the benefits and costs of moral and monetary payoffs? Here, using a neurocomputational model of decision value (DV) and functional (f)MRI, we investigated whether different brain systems are engaged when deciding whether to earn money by contributing to a “bad cause” and when deciding whether to sacrifice money to contribute to a “good cause,” both when such choices were made privately or in public. Although similar principles of DV computations were used to solve these dilemmas, they engaged 2 distinct valuation systems. When weighing monetary benefits and moral costs, people were willing to trade their moral values in exchange for money, an effect accompanied by DV computation engaging the anterior insula and the lateral prefrontal cortex (PFC). In contrast, weighing monetary costs against compliance with one’s moral values engaged the ventral putamen. Moreover, regardless of the type of dilemma, a brain network including the anterior cingulate cortex (ACC), anterior insula, and the right temporoparietal junction (TJP) was more engaged in public than in private settings. Together, these findings identify how the brain processes three sources of motivation: extrinsic rewards, moral values, and concerns for image.

## Abstract 2

Distinct brain systems are engaged when weighing whether to earn money by contributing to a ‘bad cause’ and when weighing whether to lose money to contribute to a ‘good cause,’ regardless of whether such choices are made privately or in public.

## Introduction

The brain has evolved to serve the organism’s self-survival, therefore selfishness is common in the animal kingdom. Social life, however, requires some curtailing of self-interest for the sake of effective group functioning, a behavior seen as a moral obligation in virtually every culture. Such moral actions typically come in two forms: first, a person can conform to moral values by foregoing a personal gain to avoid harming others, such as by refraining from being dishonest; second, a person may be willing to incur a personal cost to increase other people’s well-being, e.g., by donating to charities or volunteering. When moral values and monetary payoffs are at odds, such decisions involve weighing the benefits and costs of moral and monetary payoffs.

How do people choose whether or not to follow a moral course of action? Neoclassical economics suggests that people evaluate the opportunity of acting according to morals by comparing the expected material benefits and costs of a moral versus immoral action [1, 2] and then choose the action that maximizes their interests [3, 4]. Such a cost-benefit view of decision-making is at the core of the economic theory of crime, which forms the basis for most policy interventions aimed at curbing dishonesty. Along with such monetary incentives, nonmonetary motivation, such as the desire to maintain a positive social standing, has important effects on decision-making involving both monetary and moral payoffs. Furthermore, individuals may care about maintaining a positive self-image, which may require them to either fully forego the material gains that could be achieved by behaving immorally [5, 6], or to behave immorally “just enough” to maintain a positive self-image while increasing one’s payoff [7–10]. Thus, the moral payoffs associated with these decisions are often not just a function of the internal value system of the decision maker but depend also on the public visibility of these actions [11, 12]. Across many social animals, behavior is strongly influenced by whether or not actions are visible to others. Humans tend to behave more selfishly under guaranteed anonymity [13, 14] and more prosocially when observed by others [13, 15]. Recent economic theories of prosocial behavior combine heterogeneity in individual sensitivity to greed and altruism with social image concerns, i.e., the extent to which we value how others think of us [16]. In these models, motivation is three-fold: extrinsic (the material rewards associated with the action), intrinsic (the moral benefits associated with the action), and attached to image (the concerns for what others think of us). According to these models, humans exhibit preferences for dishonest or prosocial behavior not because they are intrinsically bad or good but because they weigh a mixture of these different sources of motivation.

**Fig 1:**
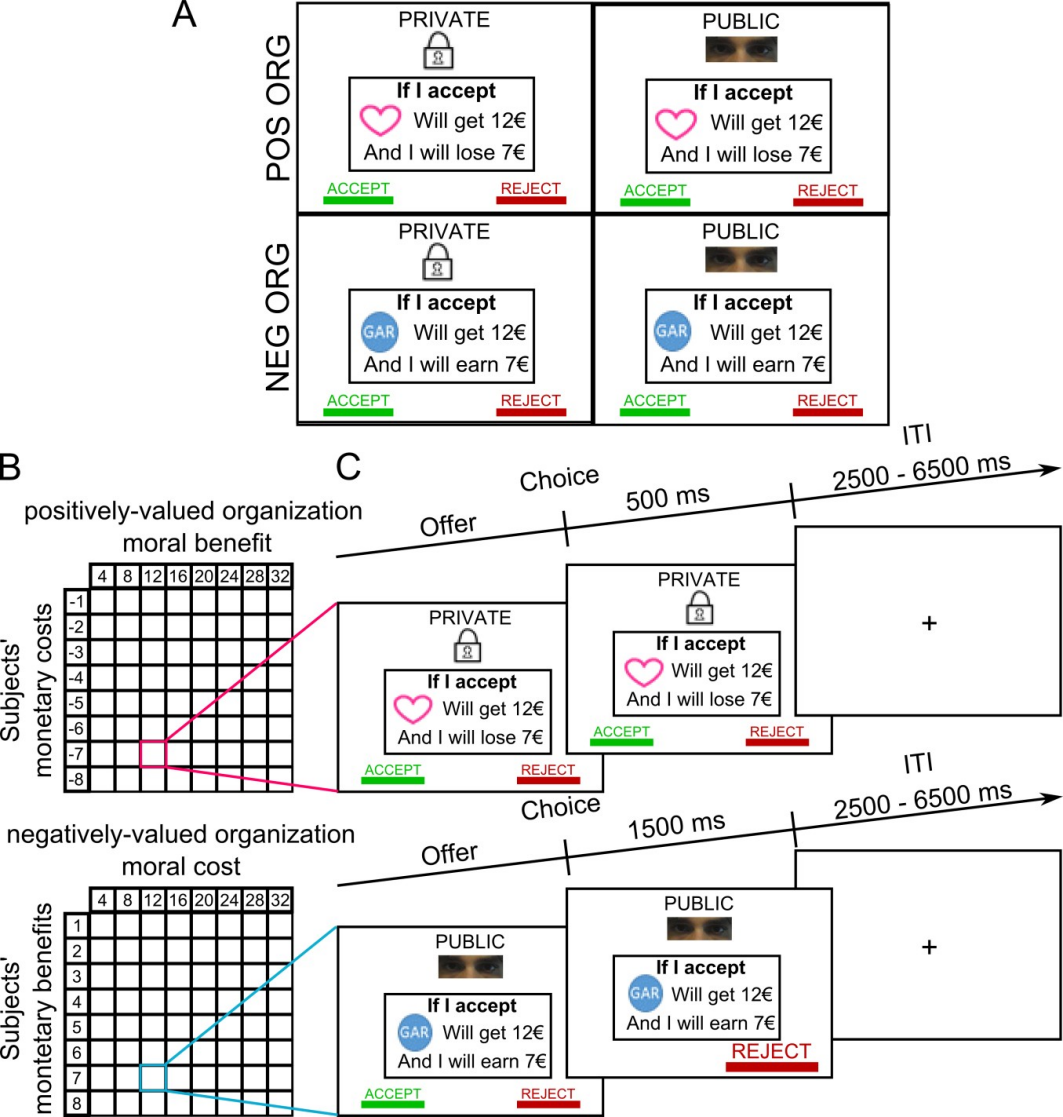
Experimental design and time course of one trial. (A) We used a 2 × 2 within-subject design, in which individuals had to accept or reject a monetarily costly action benefiting a POS ORG or a profitable action entailing a moral cost (allowing the transfer of money to a NEG ORG), either in presence or absence of observers (“public” versus “private”). (B) The amounts of the potential transfers to the organizations and of the potential costs or benefits to the subjects were varied independently across trials. In each trial, the organization potential gains ranged from 4 to 32 Euros, by increments of 4 Euros. The subjects’ potential benefits (in the NEG ORG) or costs (in the POS ORG) varied from 1 to 8 Euros, by increments of 1 Euro. This manipulation resulted in 64 different dilemmas. (C) For both the private and public conditions, each trial began with the presentation of an offer that the participant could either accept or reject by pressing a left or right button response, respectively. Then, a “feedback” screen was shown, consisting of an unchanged screen in the private condition (lasting 500 ms after choice) to keep the chosen option private (no one in the scanner room could see the choice), or in highlighting the chosen option by increasing its font for 1.5 s (while the other option disappeared) in the public condition to further emphasize the presence of observers during this condition. ITI, inter-trial interval; NEG ORG, negatively evaluated organization; POS ORG, positively evaluated organization.

Little attention has been paid to the neurocomputational mechanisms underlying brain responses in decisions weighing moral values and money. According to decision neuroscience, when choosing whether to accept or reject an offer weighing two types of attributes (e.g., moral values and money), the brain assigns a value to each option and compares them by calculating their difference. Such a scheme has been successively applied in the field of value-based decision regarding various types of benefits (e.g., money) and costs (waiting for long delay) [17–19]. Yet we know little about how the brain integrates benefits and costs into decision value (DV) when moral values and monetary gains and losses are involved. Here, we propose a neuro-computational model to shed light on how the human brain computes a DV integrating both moral values and monetary payoffs when they are at odds. We conducted a model-based functional MRI (fMRI) experiment in which participants made two types of decisions while being in the scanner: earning money by contributing to a cause they do not support and foregoing monetary gains to contribute to a cause they do support. The costs and payoffs for the participants and the two types of causes were orthogonally manipulated in order to vary the costs and benefits of behaving morally (Fig 1). The cause individuals did not support (hereafter, the “negatively valued organization”) was an existing organization negatively rated by all participants before the experiment (symbol “GAR” in Fig 1). The cause individuals supported (hereafter the “positively evaluated organization”) was a charity positively rated by all of them (symbol of a heart in Fig 1). This design allowed us to test whether separate or similar brain valuation systems are involved when facing these two organizations and their associated dilemmas, differently weighing moral values with money.

The main objective of this study was therefore to investigate whether separate brain valuation systems weigh moral costs and monetary payoffs on the one hand, and moral benefits and monetary costs on the other. Neuroimaging studies have identified a core brain network, including the ventral striatum (nucleus accumbens, putamen, or both), the ventromedial prefrontal cortex (vmPFC), and the temporoparietal junction (TPJ), engaged both when anticipating or receiving rewards and when making donations to charities [20–26]. The brain may have developed the capacity to incorporate moral considerations into its standard valuation circuitry. If this hypothesis is correct, we would expect a single valuation system to weigh both moral benefits with monetary costs on the one hand, and monetary benefits with moral costs on the other hand. Alternatively, there may be separate valuation systems for these two types of dilemmas [27]. A different system may be engaged when computing the trade-off between monetary benefits and moral costs implied by giving away one’s own moral values. Two strong candidates for computing such DVs are the anterior insula, which may treat violations of moral rules as aversive outcomes [28], and the lateral prefrontal cortex (PFC), which has often been reported to be engaged when making decisions involving dishonesty [29–31] and norm enforcement or compliance [32–35]. Manipulating systematically the cost-benefit relationships associated with monetary rewards and moral values allowed us to develop a computational account of DV trading off moral values with money, contrary to fMRI studies using classical moral dilemmas (e.g., the trolley problem) or assessing honesty based on the reluctance to lie [29, 30, 36].

Another goal of this study was to investigate the effect of an audience, i.e., the observability of choices, on the brain regions engaged in two types of decisions: allowing a monetary transfer to a charity at a monetary cost for oneself and accepting a monetary payoff at a moral cost. In our experiment, we thus varied systematically whether decisions in the fMRI scanner were made in private or could be scrutinized by an observer. Although an audience effect on prosocial behavior and on the brain circuitry engaged for a good cause has already been identified [15], it remains unknown how choices made publicly influence the brain regions engaged when deciding whether to transfer money to a charity at a personal cost and when deciding whether to earn money at a moral cost.

Using model-based fMRI, our findings revealed the existence of two distinct valuation systems operating when the decision maker faces two types of trade-off computations involving conflicting moral and monetary costs and benefits. One system centered over the bilateral anterior insula and the dorsolateral PFC (DLPFC) is engaged during valuation of a monetary transfer from a third party to the bad cause in exchange for a personal monetary gain. In contrast, a classical value-based system centered over the ventral putamen is engaged when individuals evaluate whether accepting or not a monetary transfer to a charity at a monetary cost to themselves. Moreover, consistent with a desire for social approval, we found that a brain network was more responsive when individuals acted in public than in private settings, regardless of the type of dilemma and choices.

## Results

### Behavioral results

We tested a number of models to determine how the decision process is influenced by the subjects’ monetary benefits and costs, the organization’s gains, the presence of an audience effect, the interactions between the latter factors, and time. We estimated a number of logistic regression models to identify the determinants of the participants’ choices and compared their fit to the data using the Akaike and Bayesian information criteria (S1 Table). The results of these random-effects logistic regressions are reported in S2 Table. The econometric specification that better fits the data and that—in our setting—provides the most complete account of the effect of social image on prosocial behavior is the following:

$$Pr{\left(Accept\right)}_{i}=\beta_{0}+\beta_{1}{\left(Organization\;Gain\right)}_{i}+\beta_{2}{\left(Subject’s\;Payoff\right)}_{i}+\beta_{3}{\left(Public\;Condition\right)}_{i}+\beta_{4}{\left(Organization\;Gain\times Public\;Condition\right)}_{i}+\beta_{5}{\left(Subject’s\;Payoff\times Public\;Condition\right)}_{i}+\beta_{6}{\left(Time\right)}_{i}+\beta_{7}{\left(Response\;Time\left[RT\right]\right)}_{i}+\varepsilon_{i}$$

**Fig 2:**
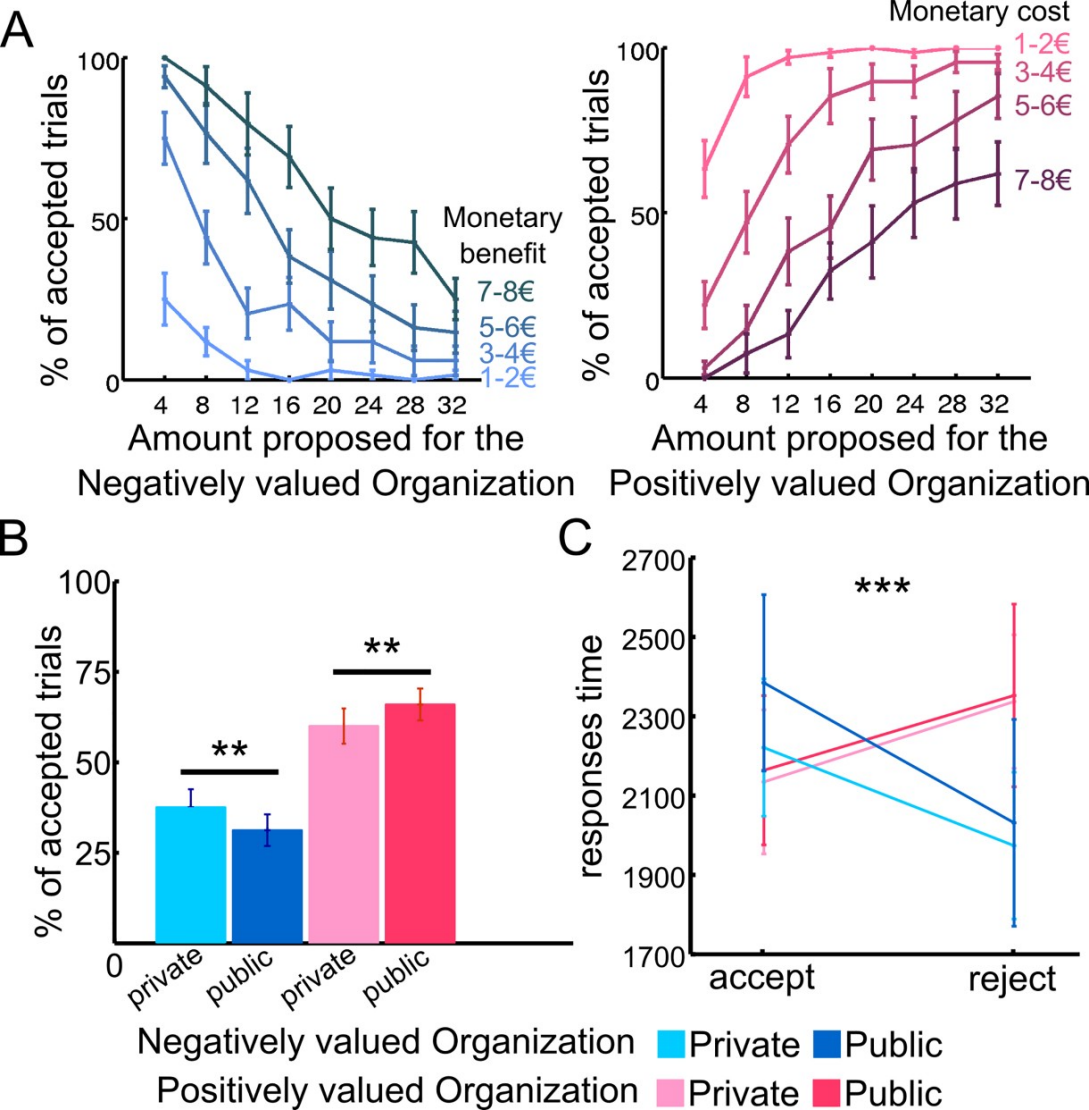
Behavioral results. (A) For the NEG ORG trials, an increase in moral costs (amount potentially transferred to NEG ORG) reduced the likelihood of a transfer being accepted, while potential monetary benefits increased this likelihood. For the POS ORG, an increase in the subject’s monetary cost decreased the likelihood of accepting the offer, while an increase in the moral benefits (POS ORG potential gain) significantly increased the acceptance rate. (B) Effect of audience on accepting offers for both organizations. For the NEG ORG, participants were more likely to accept the offers (i.e., earn money at a moral cost) in private than in public. In the charity condition, individuals were more likely to accept offers (i.e., make prosocial decisions) in public than in private. **p < 0.01. (C) RTs showing interactions between organization types and accept or reject decisions (***p < 0.001), regardless of audience/privacy effect. Participants were faster to accept transfers in the POS ORG condition and to reject them in the NEG ORG condition, suggesting opposite default strategies for the two organizations. See S1 Data. NEG ORG, negatively evaluated organization; POS ORG, positively evaluated organization; RT, response time.

When considering the negatively valued organization, an increase in the amount transferred to this organization reduced the probability of accepting the transfer, whereas the potential monetary benefit to the subjects increased this probability (Fig 2A left, S2 Table). When considering the interaction between the potential payoffs for the negatively valued organization and the public condition, we found that the presence of an audience further reduced the probability of accepting the transfer as the payoffs for this organization increased. On the other hand, the interaction between the public condition and the potential gain for the individual was not statistically significant, meaning that, all else being equal, the presence of an audience did not affect the probability of accepting or rejecting offers based on the potential gain for the subject. Note that we set up separate models for choices concerning the positively evaluated organization versus the negatively evaluated organization, because these choices were paired in our design with diametrically opposite material consequences (subject’s payoff = monetary losses versus gains, respectively). This required separate models with different regressors (resulting in parameter estimates of opposite valence).

When considering the positively evaluated organization, an increase in this organization’s gain significantly increased the likelihood of acceptance of the transfer, while an increase in the subject’s monetary cost reduced this likelihood (Fig 2A right, S2 Table). When we looked at the interaction between the public condition and the potential payoffs for the charity, the results were opposite to what we found for the negatively evaluated organization. That is, the presence of an audience further increased the likelihood of accepting a transfer to the charity as the payoffs for this organization increased. In contrast, the interaction between the public condition and the payoffs for the subject was not statistically significant. To better illustrate the acceptance rate according to each trade-off, we calculated the average acceptance rate over subjects for each proposed transfer and displayed color-coded heatmaps for each organization (S1 Fig).

We then assessed the effect of the audience for each organization. For the negatively evaluated organization, subjects were significantly more likely to reject the transfers in the public (69% of the trials, on average) than in the private condition (62% of the trials, on average, Wilcoxon signed-rank test |Z| = 2.84, *n* = 17, *p* < 0.005, Fig 2B, blue graphs). In contrast, for the positively evaluated organization, the rate of acceptance increased when decisions were made in the public condition (66% of the trials, on average) compared to the private condition (60% of the trials, on average, Wilcoxon signed-rank test |Z| = 3.08, *n* = 17, *p* < 0.002, Fig 2B, red graphs). This indicates that, regardless of the cause, subjects were more likely to choose the prosocial action in public (i.e., accept the transfer to the charity and reject it for the negatively evaluated organization). The same analysis conducted with robust standard errors and clustering at the individual level revealed the same results than the random-effect models, except that the effect of the time variable was no longer significant on the decisions regarding the negatively evaluated organization. These effects are consistent with the fact that a violation of moral norms is more likely under guaranteed anonymity, while prosocial behavior is more likely when being observed, as predicted by economic theories regarding the effect of audience on moral or prosocial decisions [13, 16].

We also performed a repeated-measures ANOVA with three factors on response times (RTs). The results of the 2 organizations (positively versus negatively valued) × 2 observability conditions (private versus public) × 2 options (accept versus reject) ANOVA revealed an interaction effect between organizations and the type of choices (F[1,16] = 16.65, *p* < 0.001) (Fig 2C and S2 Fig). Subjects were faster to respond for the prosocial option, i.e., rejecting rather than accepting the transfers to the negatively valued organization, and accepting rather than rejecting the transfers to the charity. When inspecting RTs according to the transfer range, this was particularly true for offers combining low monetary gains and high moral costs in the negatively evaluated organization and for offers combining low monetary losses and high moral benefits for the positively evaluated organization (blue cells, S2 Fig).

### fMRI results

#### Brain activity modulated by DV

We investigated the brain regions showing a correlation (either positive or negative) with DV presiding choice. When DV increases (i.e., the likelihood of accepting the transfer is higher), a positive correlation with DV indicates that brain regions show higher activity, while a negative correlation indicates a decreasing activity.

**Fig 3:**
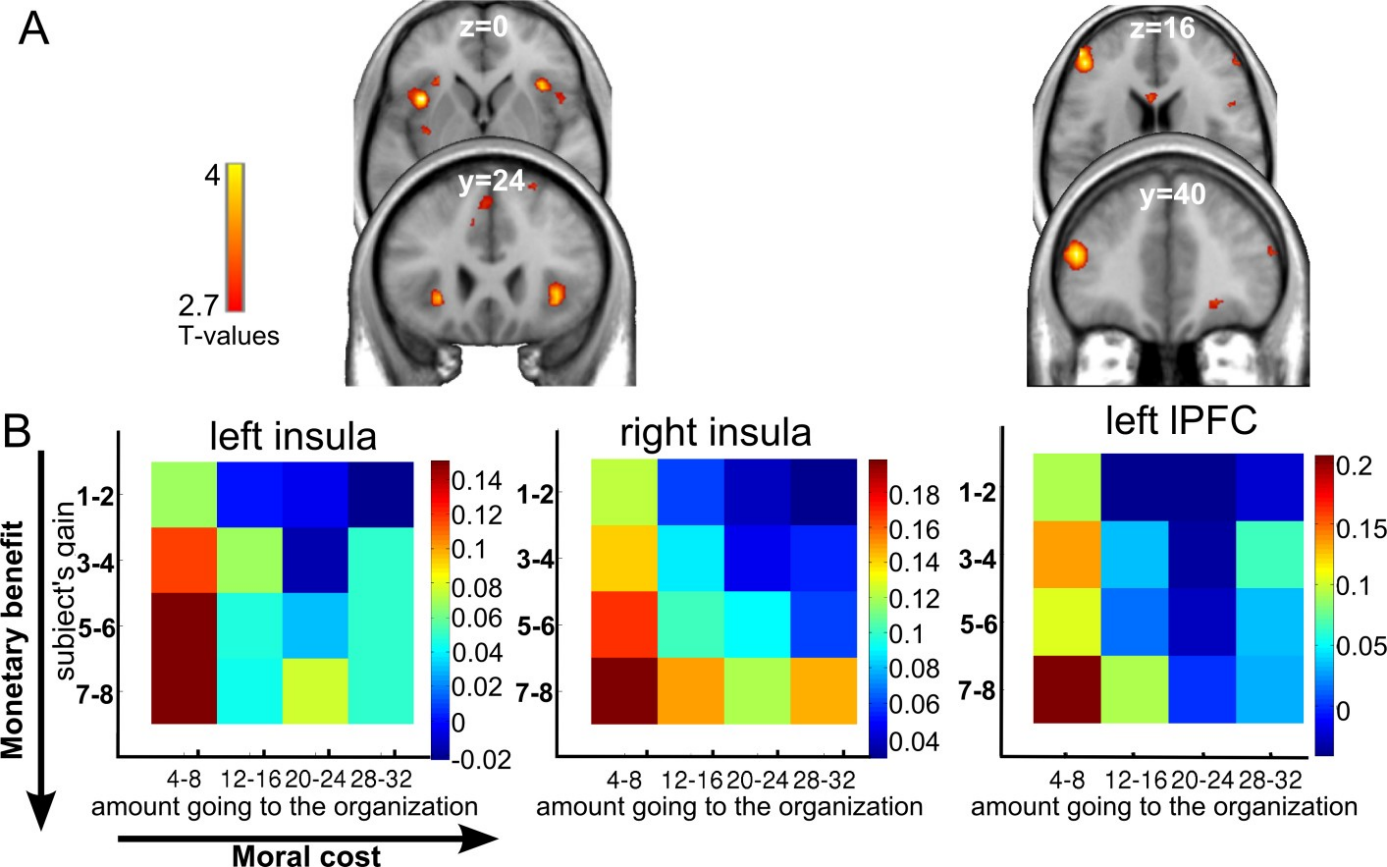
In the NEG ORG context, bilateral insula and lateral PFC activity correlated with DV at the time of decision-making. A. BOLD response in the bilateral insula and lateral PFC was positively correlated with the DV in the NEG ORG. B. Heatmaps were created by averaging parameter estimates versus baseline within anterior insula and lateral PFC clusters for each cell of a 4 × 4 monetary benefit versus moral cost matrix (resulting from collapsing the original 8 × 8 matrix, see Methods). Color coding reflects the strength of the neural response, with dark red representing the strongest activation and dark blue representing the lowest activation. See S1 Data. fMRI data corresponding to this figure can be found at the following URL: https://neurovault.org/collections/5028/. BOLD, blood-oxygenation-level–dependent; DV, decision value; fMRI, functional MRI; lPFC, lateral PFC; NEG ORG, negatively valued organization; PFC, prefrontal cortex.

First, we focused on the negatively evaluated organization and identified the brain regions showing a positive correlation between the blood-oxygenation-level–dependent (BOLD) response and DV, computed as the weighted difference between the monetary benefits minus the moral cost. This regression revealed activity in a brain network including the bilateral anterior insula (x, y, z = −36, 14, 1 and x, y, z = 36, 26, −5) and the left DLPFC (x, y, z = −48, 44, 16) (Fig 3A, S3A Table). To illustrate how the responses of the bilateral anterior insula and DLPFC contribute to the trade-off between monetary benefits and moral costs, we extracted the percent signal change (PSC) for each cell in the monetary gain versus moral cost matrix. The BOLD pattern illustrated in the corresponding heatmaps (Fig 3B) paralleled the behavioral results showing higher acceptance rate for transfers with high monetary benefits and low moral costs (see matrix of the negatively valued organization, S1 Fig). No brain region showed a negative correlation with DV in the negatively evaluated organization condition.

**Fig 4:**
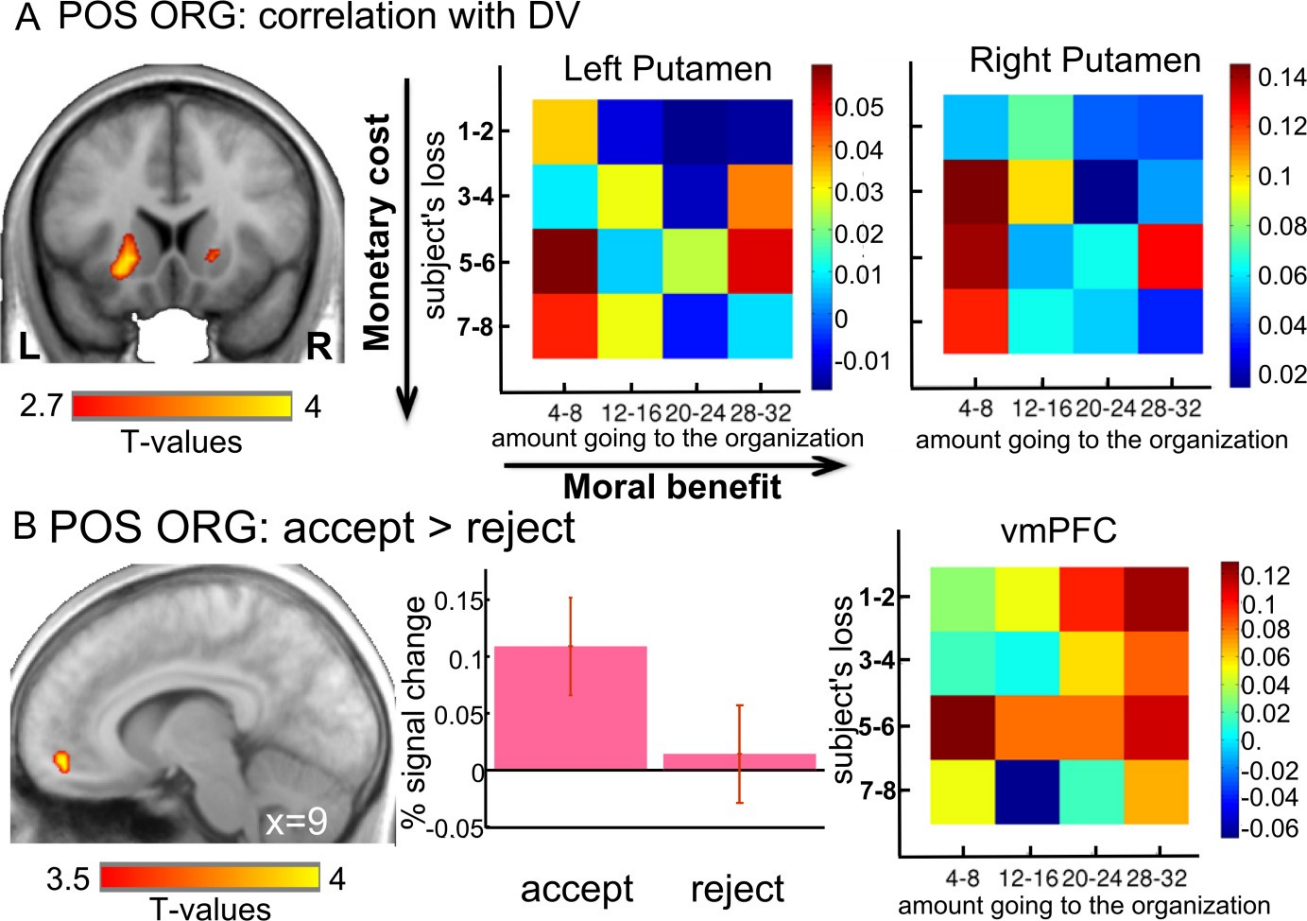
In the positively valued organization condition, ventral putamen activity correlated with DV, and ventromedial PFC was engaged with prosocial choices. (A) Activity in ventral putamen correlating with the DV in the POS ORG context. Heatmaps were created by averaging parameter estimates versus baseline within the ventral putamen clusters revealed by the DV correlation for the POS ORG for each cell of a 4 × 4 monetary cost versus moral benefit matrix (resulting from the collapsing of the original 8 × 8 matrix, see Methods). Color coding reflects strength of neural response for each condition, such that dark red represents the strongest activation and dark blue represents the lowest activation. (B) The vmPFC showed stronger activation when subjects chose the prosocial option (i.e., accept) than when they chose the selfish one (i.e., reject) in the POS ORG context. Plots of mean PSCs are shown for illustrative purpose. The color-coded heatmap was created by averaging parameter estimates versus baseline within the vmPFC cluster for each cell of the 4 × 4 monetary cost versus moral benefit matrix. See S1 Data. fMRI data corresponding to this figure can be found at the following URL: https://neurovault.org/collections/5028/. DV, decision value; fMRI, functional MRI; PCS, percent signal change; PFC, prefrontal cortex; POS ORG, positively valued organization; vmPFC, ventromedial prefrontal cortex.

Second, we focused on the positively evaluated organization and searched for brain regions engaged in DV computation. No brain region showed a positive correlation with DV (defined as the weighted difference between moral benefits and monetary costs at the time of offer) in this charity condition. Instead, mirroring the opposite behavioral pattern observed for the two organizations, a negative correlation was observed between the BOLD response and DV in the ventral putamen (x, y, z = −21, 14, −5) (Fig 4A; S3B Table). This correlation reflects that the ventral putamen showed decreasing activity as the DV increased (presiding to a higher likelihood of accepting the offer). To better understand how the activity of the ventral putamen contributes to solve the monetary benefits versus moral costs dilemma, we extracted the PSC in this functional region for each cell in the monetary gain versus moral cost matrix. The reduced BOLD signal illustrated in the corresponding heatmaps (blue cells observed for high moral benefits and low monetary cost, Fig 4A) mirrored the behavioral results of a higher acceptance rate for offers with high moral benefits and low monetary costs (red cells of the heatmaps, S1 Fig).

To ensure that the identified regions from the two valuation systems are clearly distinct, we directly compared the slope of the negative correlation with DV for the charity and the slope of the positive correlation with DV for the negatively evaluated organization. These comparisons were made in the functional clusters obtained from the DV analyses (S3 Table). Paired *t* tests were performed to compare the DV beta slope in the negatively evaluated organization condition with the DV beta slope of the charity condition, in the bilateral insula clusters positively modulated by DV in the negatively evaluated organization condition (S3A Table) and in the ventral putamen cluster negatively modulated by DV in the charity condition (S3B Table). A greater difference between slopes was found in the left ventral putamen in the charity condition compared to the negatively evaluated organization condition (t[16] = −3.57, *p* = 0.0025). Similarly, a greater slopes difference was observed in the bilateral anterior insula for the negatively evaluated organization compared to the charity (left: t[16] = −4.42, *p* = 0.0004; right: t[16] = −2.55, *p* = 0.0212). Similar significant differences in the slopes of the correlations were obtained when extracting beta from spheres around previously published coordinates (see Methods section “Activations localization and reported statistics”).

#### Brain correlates of prosocial decisions in the positively and negatively evaluated organization conditions

When investigating which brain regions correlate with prosocial decisions for the positively evaluated organization, we found that the vmPFC (x, y, z = 9, 50, −11) was more engaged when people made the prosocial decision (accepting a monetary loss to let the charity earn money) compared with choosing the selfish option (avoiding a monetary loss and foregoing the moral benefit associated to the transfer) (Fig 4B, left, S4A Table). This fMRI analysis was repeated using a new general linear model (GLM) controlling for DV, and again, we found that the vmPFC was more engaged when accepting than rejecting the offer in the charity condition. For illustrative purposes, we extracted the PSCs in this functional region, separately for the accepted and rejected trials (Fig 4B, middle). Moreover, PSCs were also extracted for each cell in the monetary cost versus moral benefit matrix, and this BOLD pattern is illustrated in the corresponding heatmaps (Fig 4B, right). In addition, in those trials when people opted for the prosocial option, an activation in the vmPFC was positively correlated with the amount transferred to the charity (S4B Table). No vmPFC activity was observed when comparing accept and reject decisions in the negatively valued organization (even at a lenient threshold of *p* < 0.005 uncorrected).

#### Effect of audience on brain activity

**Fig 5:**
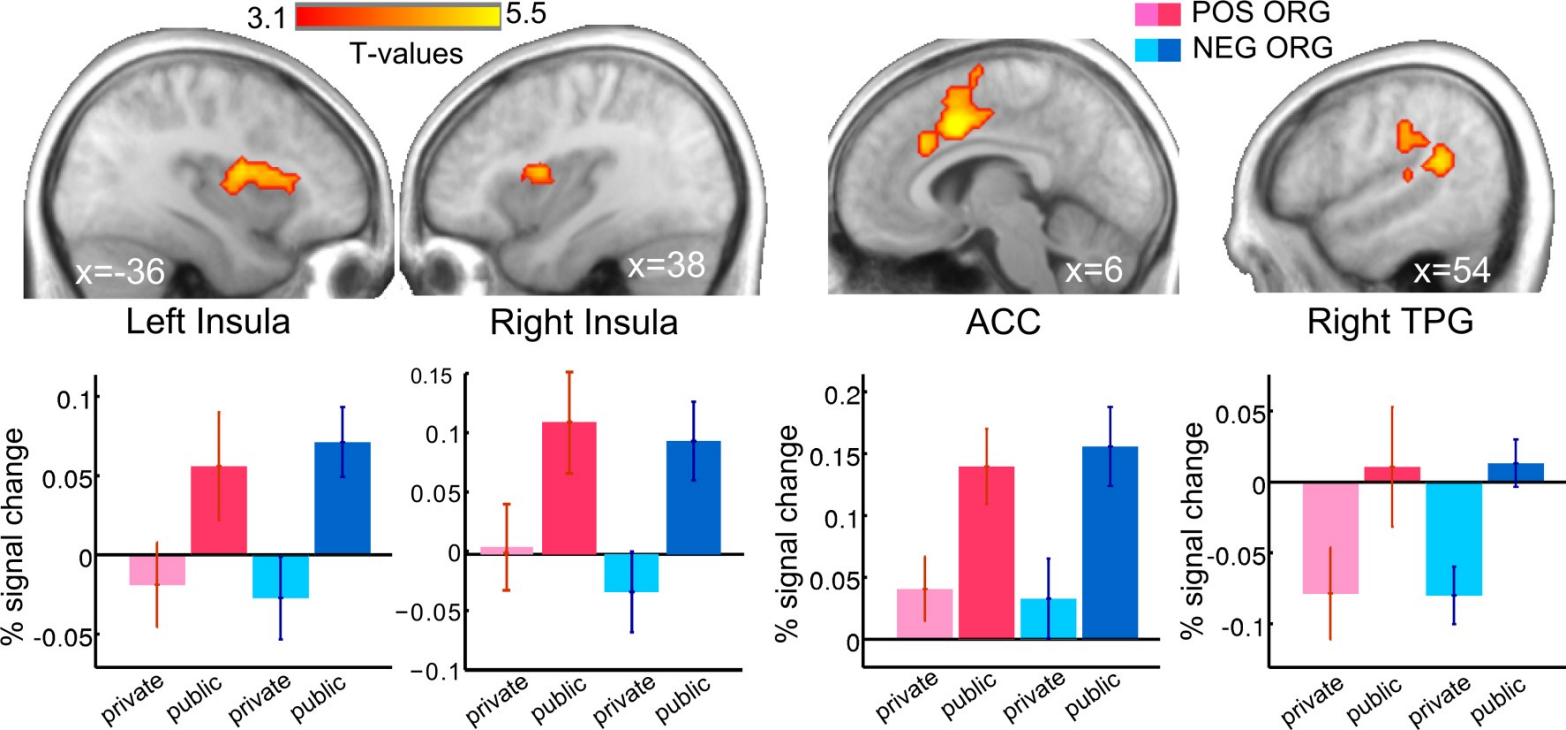
Brain responses in public relative to private. Being observed increased activity in the bilateral INS, ACC, and right TPJ. Activations are overlaid on an average anatomical scan of all subjects (cluster-wise FWE-corrected *p* < 0.05 and voxel-wise uncorrected *p* < 0.001). Plots of mean PSCs are shown for illustrative purpose. For the POS ORG and the NEG ORG, respectively, light pink and light blue represent the private trials, while dark pink and dark blue are used for the public trials. Error bars indicate SEM. See S1 Data. fMRI data corresponding to this figure can be found at the following URL: https://neurovault.org/collections/5028/. ACC, anterior cingulate cortex; fMRI, functional MRI; FWE, family-wise error; INS, insula; NEG ORG, negatively valued organization; POS ORG, positively valued organization; PSC, percent signal change; TPJ, temporoparietal junction.

We also investigated which brain regions are engaged by the presence of an audience, regardless of the actual decisions made by the subjects. We found that the bilateral anterior insula (x, y, z = −45, −4, 13; 45, 5, 7), the anterior cingulate cortex (ACC; x, y, z = 6, 8, 37), and the posterior superior temporal gyrus/TPJ (x, y, z = 48, −40, 13) were more engaged in public than in private (Fig 5, S5A Table). For illustrative purposes, PSCs were extracted in each of these functional clusters for the public and private trials, for each organization. These PSCs are plotted in Fig 5. In contrast, the bilateral inferior parietal cortex (x, y, z = −54, −49, 52 and 51, −64, 46) was more engaged when the subjects knew that they were making their decisions in private compared with when they knew they were being observed (S5B Table).

#### Correlations with single attributes

We also identified the brain regions showing a correlation with the size of the potential monetary benefit/cost and potential moral benefit/cost, using parametric regressors (S3 Fig and S4 Fig, S6 Table). In the negatively evaluated organization condition (S3 Fig), a similar set of brain regions was responsive for increasing the potential monetary gains (a positive correlation) and for decreasing the moral costs (a negative correlation with the organization’s gain). These regions included the anterior insula, the ACC, the DLPFC and the intraparietal region (S6 Table). For the charity condition (S4 Fig), a positive correlation was observed in the vmPFC with increasing moral benefits (S6 Table). No voxel survived correction for multiple comparison in the negative correlation with monetary costs.

#### Functional connectivity analyses

We performed functional connectivity analyses with DV-related brain regions (S5 Fig and S6 Fig) taking the bilateral anterior insula for the negatively valued organization and the bilateral ventral putamen for the charity as seed regions. The results revealed that the strengths of the ventral putamen-dorsal ACC (dACC) and anterior insula-dACC connectivity were positively correlated with both ventral putamen and anterior insula DV-related activity (S7 Table, S8 Table). That is, the ventral putamen-dACC connectivity strength was positively correlated with the ventral putamen DV-related activity. Similarly, the anterior insula-dACC connectivity strength was positively correlated with the anterior insula DV-related activity.

## Discussion

When faced with the opportunity of rejecting transfers to a charity to avoid a monetary loss or of making a monetary profit from a decision betraying one’s moral values, what determines people’s willingness to transgress their moral values or to comply with them? Such a dilemma may involve choosing between making money by serving a bad cause, such as evading taxes or lying, and foregoing this opportunity of increased payoffs to comply with one’s moral values. In other situations, the trade-off may be the exact opposite: individuals may have to choose between suffering a monetary loss for serving a good cause or acting selfishly. In the first case, there is a tension between pecuniary self-interest and moral costs in case of non-compliance with one’s moral values, whereas in the second, there is a tension between the monetary loss and the moral benefit in case of compliance with one’s moral values. Here, we demonstrate that such trade-offs engage two separate valuation systems but that both systems perform similar computations. The anterior insula together with the lateral PFC compute the DV (i.e., the difference between the subject’s monetary benefit and the associated moral cost weighted by the absolute value of the individual regression coefficients from the selected logistic model), implementing the trade-off between monetary benefits and moral costs (Fig 3). In contrast, the ventral putamen computes the DV (the difference between the subject’s moral benefit and the monetary cost weighted by the absolute value of the individual regression coefficients) related to the trade-off between moral benefits and monetary costs (Fig 4A). These findings indicate that similar computational rules are applied by distinct brain systems, depending on the direction of the benefits or costs for the subject and the direction of the moral values. In addition, consistent with the view that moral benefits have intrinsic value [26, 37], the vmPFC was sensitive to higher moral benefits (Fig 4B). The medial PFC has also been reported to be active during social referencing and mentalizing about others [38, 39].

Our study demonstrates the existence of two separate valuation systems when computing DVs for different types of cost/benefit trade-off between moral values and money. Such findings are consistent with an early theoretical proposal suggesting that there may be separate valuation systems for two types of considerations: one treating violations of moral norms as aversive outcomes, and another treating compliance with moral rules as a rewarding outcome [27]. One possible mechanism explaining why distinct valuation systems were observed in the computation of DV for the two organizations is that they required anticipation of outcomes as rewards (doing good) or penalties (doing bad)—known to recruit, respectively, the ventral putamen/vmPFC and the bilateral anterior insula/lateral PFC [40–43]. The engagement of the anterior insula is observed when being socially excluded [44], when being treated unfairly [45], during anticipation of risky losses, and when making inequitable decisions [26]. The DLPFC is engaged during trade-offs between behaving honestly and the pursuit of self-interest [29–31] and by monetary gains made from harming others, but not self [46]. In contrast, fMRI studies on social rewards report engagement of the ventral striatum with good reputation, being treated fairly, and being cooperative [15, 26, 47].

The current study adds to this body of work by demonstrating that the anterior insula/lateral PFC and the ventral putamen are not only engaged by negative and rewarding social events per se, respectively. They perform neural computation of different types of DVs: difference between monetary benefits and moral costs on the one hand, and between moral benefits and monetary costs on the other hand. That is, in our design, these brain regions computed a value difference preceding choice behavior, extending results from value-based decision-making [17, 18]. However, in some of these previous fMRI studies, one choice option was kept constant [18], precluding these studies from distinguishing between valuation and value comparison processes because the value of the changing option correlated with the value difference between options. In contrast, in the current study, on each trial a different trade-off between benefit and cost was at stake, allowing us to compute a DV reflecting a true value difference. Therefore, our results show that the anterior insula/DLPFC and the ventral putamen are able to weigh on the same scale different costs and benefits of different nature in the moral and the monetary domains.

Recent behavioral economics experiments have provided evidence that when people face the opportunity to act unethically, they often do so but only to a certain extent in order to maintain a positive self-image [7, 9, 10]. Our behavioral results are consistent with these findings in a different environment by showing that subjects are willing to trade their moral values for monetary benefits (i.e., they accept transfers entailing a moral cost in exchange for a monetary benefit) but only when the moral cost is not too high (Fig 2A, left). Our behavioral findings are consistent with nonstandard economic models proposing that immoral actions originate not in rational self-interest per se but in affective responses to social behavior [16, 48]. According to this view, social principles—such as image motivation—have an intrinsic value. Although individuals undoubtedly value a personal monetary gain, they also display an aversion to betraying their moral values. Thus, people have to strike a balance between two motivating forces, such that they derive some financial benefits from behaving dishonestly or violating a norm but still maintain their positive self-image.

Our behavioral results also show that subjects were more likely to accept giving to the charity at low monetary costs to themselves, and were faster to do so, but they were more likely to reject transfers to the negatively valued organization for low gains to themselves, and again, were faster to do so (Fig 2A and 2C and S1 Fig, S2 Fig). Thus, the choice and RT behavior clearly indicates opposite default strategies for the two organizations, suggesting that participants internalized different a priori default preferences for the two organizations. That is, the default strategy was to accept the transfer to the charity, perhaps engaging approach behavior and appetitive processes, whereas for the negatively valued organization, the default strategy was to reject the transfer, likely to engage avoidance behavior and aversive processes. This interpretation parallels recent debates about the framing of DV signals, which may reflect computation of a relative value difference between the a priori default option and the alternative option [49]. This pattern of behavioral results (choice and RTs) reflecting opposite appetitive or aversive processes may explain why we observed brain regions showing decreasing activity with higher DV in the charity condition and increasing activity with increasing DV for the negatively valued organization. In particular, in the charity condition, when the correlation between the BOLD signal and DV decrease (blue cells of Fig 4A), there is a rapid accept judgment for cells corresponding to low subject losses and high amounts to charity (see S1 Fig and S2 Fig). Conversely, for offers combining high personal monetary losses with low moral benefits, higher ventral putamen activity may reflect that successful avoidance of the offer may be, in itself, rewarding and engage similar neural mechanisms than reward processing. Such involvement of the putamen in active avoidance mechanisms has been previously observed in both nonhuman [50–54] and human studies [55–59]. This may explain why our ventral striatum findings engaged the ventral part of the putamen rather than the nucleus accumbens. Also, it should be noted that the negative correlation between ventral putamen activity with DV observed in the charity condition (Fig 4A) does not mean that the response of this brain region increases with higher personal costs and with decreasing benefits to the charity, as demonstrated by the single attributes analyses (S4 Fig).

Our findings extend the distinction between two brain valuation systems identified in recent years to monetary and moral costs/benefits. For example, separate valuation systems for delay (anterior insula and dACC) versus effort (ventral striatum/vmPFC) cost/benefit decisions have been reported in both rodents and humans [60]. Similar evidence coming from a combined Electroencephalography (EEG)-fMRI study points to the existence of distinct decision-outcome value systems that can be dissociated temporally [41]. The dichotomy between appetitive and aversive processes differently engaged by the 2 valuation systems may also explain why distinct networks have been observed in empathic care and distress [61, 62]. Empathic care, which leads to helping behavior, has been associated with appetitive processes engaging the ventral putamen, whereas empathic distress, which leads to avoidance behavior, has been associated with aversive processes engaging the anterior insula [40, 61].

It remains unclear whether different attributes (moral benefits and monetary costs on the one hand, and monetary benefits versus moral costs on the other hand) are represented in the same valuation systems that perform the weighted difference between them, or whether these attributes are represented in neural structures distinct from those in which the comparison is performed. When investigating the neural implementation of these attributes for the negatively valued organization, we found that a similar brain network, including the bilateral anterior insula, dACC, and DLPFC, was engaged both for increasing monetary benefits and for decreasing moral cost (S3 Fig, S6 Table). Consistent with this effect, the anterior insula has been associated with both positive and negative correlations with subjective value in a conjunction meta-analysis [40], reflecting a quantity such as salience [63, 64]. For the charity, the correlation between moral benefit attributes and the BOLD signal showed engagement of the vmPFC (S4 Fig). Prior research strongly implicates the medial PFC when making altruistic choices [22, 23, 26, 65], although its precise computational role remains debated [66]. Thus, for the negatively valued organization, the anterior insula represented both single attributes and their weighted differences, while for the charity, the ventral putamen integrated single attributes in the DV signal. In addition, to investigate whether the DV signals computed in separate neural systems for the 2 types of trade-offs are passed to common brain regions, we performed seed-to-voxel functional connectivity analyses taking DV-related activity from the anterior insula for the negatively valued organization and the bilateral putamen for the charity as seed regions (S5 Fig and S6 Fig). This analysis provided direct evidence that both identified valuation systems functionally interact with the ACC, possibly reflecting an integration of decision parameters [67, 68]. Although the connectivity analysis did not reveal direct relationships between the ventral putamen and the vmPFC for the charity condition, the strength of the coupling obtained from resting-state functional connectivity between the vmPFC—observed in cost-benefit valuation—and the putamen/caudate nucleus has been shown to be strong, both in primates and nonhuman primates [69].

The presence of an audience reduced the likelihood of accepting the transfer as the payoff for the negatively evaluated organization increased, while it increased the chance of accepting a transfer to the charity as the payoffs for this organization increased. At the brain system level, for both organizations, the presence of an audience (public versus private choices) engaged a brain network including the anterior insula, the ACC, and the right TPJ (Fig 5). The engagement of this brain network with concerns for social image may reflect meta-representations required for representing what other people think of us [36], such as the desire to conform to moral norms while giving to charities or when refusing to give to a bad cause [70–73]. Yet a recent Transcranial Magnetic Stimulation (TMS) study indicates that the right TPJ may not be necessary to react to social reputation cues but may instead reduce the behavioral impact of moral-material conflicts [74], illustrating the need for noninvasive brain stimulation to establish the causal role of a specific brain region in a given function [75].

Previous neuroimaging studies on moral reasoning have relied on paradigms that involved judging actions from a third-party perspective [76] and judging highly hypothetical and often extreme moral dilemmas (e.g., killing one person to save the lives of many, see [77]). In real life, however, individuals are repeatedly faced with more ecologic dilemmas in which moral and monetary stakes vary; as such, it is critical to understand how the brain computes the DV presiding choice when weighing moral costs and monetary benefits. Other studies related to moral choices have investigated decisions involving dishonesty and lying [29–31], decisions whether to comply to social norms [32, 34, 35, 78], decisions involving a trade-off between money and physical pain [46, 71, 79], and decisions about moral and religious statements [80, 81]. Yet, our study is the first to combine, in the same design, the investigation of the neural correlates of the trade-off between accepting money for moral violations and those engaged in weighing whether to accept losing money for the benefit of a charity.

One potential limitation of our findings is that it only concerns men. Future studies should investigate whether the current findings extend to women too. We chose to scan only men because gender has been shown to affect prosocial behavior [82, 83] and unethical behavior [84, 85]. Moreover, young women experience hormonal modulations of the reward system [86], which may affect the neural substrates of the two brain systems investigated in the current study. In addition, there are known interactions between the effects of audience and the observer’s gender (kept constant in the current experiment). For example, in women the mere presence of men can induce transient decrements in cognitive efficiency and academic performance when confronted with math tests despite similar performances when tested separately [87, 88].

## Conclusion

Our study provides evidence that, consistent with models integrating monetary motivation, image concerns, and compliance with moral values [7, 10, 16, 89], people are willing to bend their moral values to earn more money, but only to a certain extent, an effect engaging the anterior insula and the lateral PFC. The ventral putamen computes a weighted difference between moral benefits and monetary costs, rather than reflecting these attributes in isolation, while the vmPFC is more engaged with moral attributes alone and with prosocial choices for the charity. Moreover, consistently with models of social behavior influenced by a desire for social approval, we found activity in a social brain network that was more responsive in public than in private settings, regardless of whether the individual supports the cause or not. A deeper understanding of how the brain weighs moral values, monetary payoffs, and social image when people are asked to refrain from immoral acts or are encouraged to undertake moral actions may help in the design of new types of policy interventions. Indeed, in the last two decades, governments and social scientists have become increasingly interested in using social nudges, such as moral suasion [90, 91] and social recognition to deter antisocial behaviors and promote prosocial activities. A better understanding of the neural mechanisms at stake may participate in identifying in which conditions these interventions are more likely to be effective.

## Methods

### Ethics statement

The study was approved by the local ethics committee (Comité de Protection des Personnes SUD-EST IV, autorisation n° 22036S, DGS2008-0179). This experiment adhered to the Declaration of Helsinki.

### Subjects

Twenty-four healthy subjects (all men, age = 22.47 ± 2.62 years) with no history of neurological or psychiatric illness participated in the fMRI experiment. Two subjects were excluded from the analysis for technical imaging problems. All subjects were right-handed, as assessed by the Edinburgh Handedness Inventory, and presented no symptoms of depression, as assessed by the 13-item version of the Beck Depression Inventory. All subjects gave written informed consent to be part of the experiment, which was approved by the local ethics committee (CPP Centre Léon Bérard, Lyon, France).

### Pretesting

Before the fMRI experiment, a behavioral pilot study involving 48 healthy volunteers was carried out at GATE-Lab, Lyon, to help in designing stimuli and task procedures. To guide the selection of the organizations, we asked these participants to complete a questionnaire after the presentation of brief descriptions and logo images of 14 organizations. Organizations with positive or negative valence were presented. For each one, participants had to rate their feelings towards them on a scale from −10 to 10. The order of the organizations in the questionnaire was randomized across subjects. Based on this pilot study, we chose for the fMRI experiment the two organizations that received the worst (mean = −5.73, SD = 3.68) and the best (mean = 8.40, SD = 2.04) ratings. Because *PLOS Biology* policy does not allow us to publish trademarked names, we have changed the real names of these two organizations. GAR represents the negatively evaluated organization, and a symbol of a heart represents the positively evaluated organization (a charity providing food to homeless and poor people).

### Experimental task

We used a 2 × 2 within-subject design, in which individuals decided whether to accept or reject monetary transfers to the two organizations. In some blocks of decisions, the subjects had to accept or reject offers of transfers to the positively evaluated organization. In other blocks of decisions, offers concerned the negatively evaluated organization. Decisions were made either in presence or absence of observers (“public” versus “private” conditions) (Fig 1A). At the beginning of the experiment, subjects received an initial endowment of 14 Euros. During the experiment, they were faced with successive offers involving a variable monetary payoff for themselves and a variable payoff for the organization. When making decisions regarding the positively evaluated organization, subjects had to decide whether to accept or reject monetary transfers by the experimenter to the organization at a variable monetary cost to themselves, deducted from their initial endowment. When making decisions regarding the negatively evaluated organization, they had to decide whether to accept or reject monetary transfers by the experimenter to the organization in exchange for a personal monetary payoff added to their initial endowment. In the latter case, the only way for a subject to earn money was to allow the experimenter to transfer money to the negatively evaluated organization, whereas in the former treatment, any transfer to the positively evaluated organization involved a monetary loss for the subject. One important aspect of our experimental design is that in both treatments, each organization would receive a monetary gain; in one case, however, such a gain reflected a moral cost to the individual (sending money to a negatively evaluated organization to earn money for oneself, i.e., non-compliance with one’s moral values for money), while in the other case, the gain to the organization reflected a moral benefit for the individual (altruistically foregoing a personal gain to benefit a positively evaluated organization, i.e., complying with one’s moral values). Because we systematically varied the monetary cost of a moral decision, we were able to identify the price elasticity of demand for moral actions. Intuitively, if participants did not perceive such actions as immoral, they would display no elasticity to the moral cost of choosing the self-serving action.

The monetary stakes for the organizations and for the subjects varied independently across trials. In each trial, the organization’s potential gains ranged from 4 to 32 Euros, in increments of 4 Euros. Subjects’ potential payoffs (in the case of the negatively evaluated organization) or costs (in the case of the positively evaluated organization) varied from 1 to 8 Euros, in increments of 1 Euro. Each subject was therefore exposed to 64 different dilemmas (Fig 1B).

To guarantee the independence of each decision, only one public decision and one private decision among all the trials were randomly selected for payment at the end of the experiment. If the subject accepted the offer in the randomly selected trial, the amount of the accepted transfer was sent to the organization (the mean of the two amounts was used if the two trials concerned the same organization), and the subject’s endowment was increased or decreased based on his decision. If the same organization happened to be randomly selected twice, then the organization received the average transfer and the subject’s endowment was adjusted based on the average of the two decisions. If the subject rejected the offer in the randomly selected trials, nothing was sent to the organization, and the subject’s initial endowment was not modified.

The presence or absence of an observer (public versus private conditions) was displayed on the screen in the following way. In private trials, a yellow frame surrounded the offer, and a picture of a padlock was displayed at the top of the screen reminding subjects about the privacy of their decision. In the public condition, a cyan frame surrounded the offer, and a picture of the eyes of an observer was displayed above, reminding participants that an independent observer would see their decisions. Indeed, cues of being watched exert an influence on subjects’ behavior [15]. To further stress the visibility of their choices in the public trials, participants knew that an observer in the control room, to whom they were introduced prior to the experiment, would see the subject’s screen and therefore observe their public trials decisions; in the public trials, the chosen alternative was highlighted for 1.5 s on the screen by expanding the font, while the other option disappeared. In the private condition, no changes were made on the screen after the response, assuring subjects that nobody would be able to see their choices from the scanner control room. Finally, at the end of the experiment, subjects had to declare in front of a video camera which decision they made in the randomly selected trial for the public condition. Subjects were told that decisions in the private condition were recorded anonymously, guaranteeing that none of the experimenters could link a subject’s identity with his decisions. A person not affiliated with the experiment and unaware of its content paid all subjects. All the subjects reported believing in the manipulation.

For each possible combination of individual and organization payoffs, and for both organizations, participants made two decisions, one in private and one in public. Participants therefore made a total of 256 decisions, 128 related to the negatively evaluated organization and 128 related to the positively evaluated one. Each trial began with the presentation of an offer, which could either be accepted or rejected by pressing the left or right button on a response pad (Fig 1C). A fixation cross was displayed during a random time interval (jitters), drawn from a uniform distribution between 2.5 and 6.5 s. Subjects were encouraged to make their decision within 3 s. After this delay, a message was displayed on the screen to remind them to respond.

The scanning session was divided into 4 runs of 64 trials. The first 2 runs concerned one organization and the last 2 concerned the other organization. Within the first run of each organization, the first half of the trials was either public or private, with the opposite for the subsequent run. The order of the private/public conditions in the second run mirrored the order of these conditions in the first run. The order of presentation of the organizations and of public/private conditions was balanced across subjects. Thirty-two dilemmas from the 64 possible combinations were presented in each run and each private/public condition. To guarantee that the 2 pairs of runs of each organization were balanced with respect to the payoffs for the individual and the organization, we assigned to one run the set of dilemmas composed by the subject’s odd potential payoffs and the 4, 12, 20, and 28 potential amounts for the organization, while the other run was assigned the 32 remaining dilemmas of the matrix. Within this criterion, the order of the 32 dilemmas was randomized.

Visual stimuli were back-projected on a screen located at the head of the scanner’s bed and presented to the participants through an adjustable mirror located above their head. The presentation of the stimuli was controlled by Presentation software (Neurobehavioral Systems), which also recorded trigger pulses from the scanner signaling the beginning of each volume acquisition.

### Procedures

During a first interview, participants were asked to rate their feelings toward each of 14 organizations on a scale ranging from −5 to 5. For the fMRI experiment, we selected only participants who rated the positive organizations with a score greater than 0 and the negatively evaluated organizations with a negative score (only 1 subject was excluded based on the initial rating). The day of the experiment, subjects first received instructions about the experiment. To guarantee the independence of each choice during the experiment, subjects were instructed that only one public decision and one private decision among all the trials would be randomly selected for payment at the end of the experiment.

After receiving the instructions, subjects did a few free practice trials of all conditions in the control room of the fMRI and were allowed to ask questions. After the practice session, subjects were asked to read a description of the two organizations. Before entering the fMRI room, they met with the independent observer. After scanning, the subjects were debriefed. Participants filled a post-experimental questionnaire asking whether they truly perceived the different trials as independent, whether they believed in the difference between private and public conditions, and whether they thought that the presence of the observer had influenced their decisions. Participants were then placed in front of a computer that randomly drew two trials, one public and one private. To honor the privacy condition, only the decision made in the public trial was displayed on the subject’s computer screen, while the private decision was directly sent to a person unaffiliated with the experiment and unaware of its content. Finally, for the selected public trial, subjects had to declare the payoffs for them and the organization while being filmed by an experimenter with a video camera.

### fMRI data acquisition

fMRI data were acquired on a 1.5 T Siemens MRI scanner. Scanning was divided into 4 sessions. BOLD signal was measured with gradient echo T2* weighted echo-planar images (EPIs). Twenty-six interleaved slices parallel to the AC-PC line were acquired per volume (matrix 64 × 64; voxel size = 3.4 × 3.4 × 4 mm; TR = 2,500 ms; TE = 60 ms). We used a manual shimming within a rectangular region including the orbitofrontal cortex and the basal ganglia to improve the local field homogeneity. A high-resolution T1-weighted structural scan was acquired for each subject (matrix 256 × 256 × 176; voxel size = 1 × 1 × 1 mm; TR = 1,970 ms; TE = 3.93 ms; flip angle = 15).

### fMRI preprocessing

Data were preprocessed and analyzed using the SPM8 software package (Wellcome Department of Imaging Neuroscience, London) implemented in Matlab 7.7 (Mathworks, Natick, MA). We removed the first 4 functional volumes of each session to allow the BOLD signal to reach a steady state. The remaining images were spatially realigned and unwarped in order to correct for motion artifacts. Unwarping was performed based on phase maps calculated using the Fieldmap SPM toolbox. Then, to suppress the residual fluctuations due to interpolation errors from large motions, the motion adjustment algorithm provided in the ArtRepair toolbox was used after smoothing with a 4 mm full width at half maximum (FWHM) Gaussian kernel (https://cibsr.stanford.edu/tools/human-brain-project/artrepair-software.html). This method is an alternative to add motion regressors to the design matrix. The scan artifacts were then detected and repaired using both global intensity and scan-to-scan movement with the Artifact Repair algorithm from the ArtRepair SPM toolbox [92].

For each participant, the structural image was coregistered to the mean functional image, segmented into white and grey matter, and the grey matter was normalized to a standard grey matter template. The transformation parameters estimated in this step were applied to all functional images. Functional images were spatially smoothed with a 7 mm FWHM Gaussian kernel, and finally, the structural images were averaged across subjects to create a mean image for display purposes.

### Behavioral analysis

To examine the relationships between the parameters of the tasks and subjects’ decisions, we have estimated several random-effects logistic models at the group level, one for each organization (see S2 Table). We used random-effects specification because the same subjects made 256 decisions each (conducted with software Stata version 14.2). Based on the goodness of fit of these behavioral models to the data, we selected the best model reporting the probability of accepting a transfer to an organization as a function of the payoff for the subject (which, by design, was positive for the negatively evaluated organization and negative for the positively evaluated organization), the potential gain for the organization, the private/public condition, and the interaction between the public/private condition and the payoffs for the participant and for the organizations. We also included a time trend (trial number) as an independent predictor to control for a potential influence of previous decisions, as well as RT. The following model was estimated:

$$Pr{\left(Accept\right)}_{i}=\beta_{0}+\beta_{1}{\left(Organization\;Gain\right)}_{i}+\beta_{2}{\left(Subject’s\;Payoff\right)}_{i}+\beta_{3}{\left(Public\;Condition\right)}_{i}+\beta_{4}{\left(Organization\;Gain\times Public\;Condition\right)}_{i}+\beta_{5}{\left(Subject’s\;Payoff\times Public\;Condition\right)}_{i}+\beta_{6}{\left(Time\right)}_{i}+\beta_{7}{\left(RT\right)}_{i}+\varepsilon_{i}$$

where *i* denotes the current trial.

This model was also estimated with a simple logit model with robust standard errors and clustering at the individual level to control for serial correlation within each individual. Because the results of the estimation with random effects are not qualitatively altered, we only report the results of the initial estimations.

Next, individual logistic regressions were conducted separately for each participant (collapsing over scanning runs) using the same model as above, using MatLab 7.7 (MathWorks, Natick, MA). The individual regression coefficients obtained from these regressions for each organization’s gain β_1_(subject) and the subject’s payoff β_2_(subject) were then used to compute the DV corresponding to each offer (see next paragraph on DV computation).

### DV computation

There is a consensus in decision neuroscience that individuals make decisions by assigning values to different options, taking into account the benefits and the costs associated with each option and weighing them on a common scale. The DV refers to the net value of a specific decision option that is under consideration by the agent, usually computed by weighted difference between the benefits minus the costs [37, 93]. During our experiment, subjects made multiple decisions by weighing the amount proposed for the organization and the amount they could gain or lose. For the negatively valued organization, the subject’s payoff is a monetary benefit, while the organization’s gain can be considered a moral cost to the subject. In contrast, for the positively valued organization, the organization’s gain can be considered a moral benefit, while the subject’s payoff represents a monetary cost. Therefore, for each offer, DV depends upon the organization gain and the subject’s payoff and on how each participant subjectively weights these two variables according to his subjective preferences. The individual regression coefficients β_1_ and β_2_ obtained from the individual logistic regressions described above reflected how subjects weighted the organization gain and the subject’s payoff in the DV calculation. DV was therefore determined by calculating the difference between the subject’s payoff and the organization’s gain weighted by the absolute value of the individual regression coefficients: β_2_(subject) and β_1_(subject), respectively.

For the negatively valued organization, DV was computed as the difference between the subject’s monetary benefit and the moral cost weighted by the absolute value of the individual regression coefficients: β_2_(subject) and β_1_(subject), respectively:

$$DV\left(s,i\right)=\beta_{2}\left(o,s\right)\times monetary\;benefit\left(i\right)-\left|\beta_{1}\left(o,s\right)\right|\times moral\;cost\left(i\right)$$

where *i* is the current trial and *s* is an index for a given subject.

Concerning the positively evaluated organization, DV was computed as the difference between the subject’s monetary cost and the moral benefit weighted by the absolute value of the individual regression coefficients: β_2_(subject) and β_1_(subject), respectively:

$$DV\left(s,i\right)=\beta_{1}\left(o,s\right)\times moral\;benefit\left(i\right)-\left|\beta_{2}\left(o,s\right)\right|\times monetary\;cost\left(i\right)$$

where *i* is the current trial and *s* is indexing a given subject.

This definition of DV is consistent with the value-based decision-making literature in which DV is defined as a weighted difference of benefits and costs. With these two DVs, for both organizations, subjects were more likely to accept the offer when DV was positive and more likely to reject it when DV was negative. Thus, the higher the DV, the higher the likelihood the subject accepted the offer. DV values were entered in the fMRI model at the time of the offer to identify the brain regions showing a modulation of the BOLD response. Five subjects were excluded from the DV analyses because they always chose the same option for at least one organization.

### fMRI data analysis

At the single-subject level, statistical analyses were performed using a GLM in which all regressors were modeled as delta functions and convolved with a canonical hemodynamic response function (HRF). We applied a high-pass filter with a cut-off of 128 s to the time series to remove low-frequency noise and baseline drifts, and we used an AR(1) model plus white noise to correct for temporal autocorrelation. Estimations were done in an explicit grey matter mask based on the tissue probability map provided by SPM. Because this study was designed to answer several questions, different analyses were performed to address them.

### Brain activity modulated by DV

The first GLM (GLM1) was designed to distinguish brain regions modulated by the DV. In this model, the “offer onsets” and the “subjects’ response onsets” were modeled as separate events, each divided into 4 regressors according to the condition: 2 (private versus public) **×** 2 (positively versus negatively evaluated organization). The 4 regressors of the “offer onsets” were modulated by two additional orthogonal parametric regressors: (a) the DV and (b) the RT. The 4 “response onset” regressors were modulated by the subject’s choice (1 for accept, −1 for reject). Then, subject-specific parameter estimates of the DV regressors were entered in a second-order within-subject factorial analysis with 2 organizations (positively versus negatively evaluated) **×** 2 audience (private versus public) conditions. (Note that because there was only 1 organization per run, this resulted in only 2 “offer onset” regressors per run.) We used this factorial analysis to perform 2 main comparisons in order to identify the brain regions engaged in the trade-off between (a) moral cost and self-interest benefit for the negatively evaluated organization (i.e., showing a positive correlation with the DV, computed as the monetary benefits minus the moral costs) (Fig 3A) and (b) moral benefit and monetary cost for the positively evaluated organization (i.e., showing a negative correlation with DV computed as the moral benefits minus the monetary costs) (Fig 4A). The DV results from Figs 3 and 4 show the public and private conditions combined together. Note that we chose to have separate public/private regressors rather than running another GLM modeling the private and public trials in a same regressor itself modulated by a new DV integrating the audience effect. Indeed, this would not be a parsimonious hypothesis because this would make the following assumptions: (i) that the decision process is exactly identical in the private and the public conditions (which may not be the case) and (ii) that some brain regions compute a DV integrating, all together, moral value, monetary incentive, and audience. Keeping the classical definition of DV as a weighted difference between benefit and cost also allowed us to compare our results to the large value-based decision-making literature using this definition.

Additionally, PSCs were extracted in the functional ROIs using the MarsBaR toolbox (**http://marsbar.sourceforge.net/**), from a new GLM in which the “offer onsets” were split into 16 separate regressors corresponding to the 8 **×** 8 dilemma matrix (Fig 1B) collapsed into a 4 **×** 4 matrix, for each organization and each audience condition. These PSCs were averaged over subjects to create colored-coded heatmaps for each dilemma (Figs 3B and 4).

### Prosocial decisions and audience and/or privacy effects

A second model (GLM2) was used to identify a number of brain regions, such as those associated with making prosocial choices in the charity condition and those engaged with an audience effect regardless of organization types or choice. This model included 8 regressors of interest at the time of “offer onset” in separate conditions 2 (accepted trials versus rejected trials) × 2 (private versus public) × 2 (positively versus negatively evaluated organization). We included the size of the potential gain for the organization and the size of the potential gain or loss for the subject with 2 orthogonal parametric regressors. The subject’s response was modeled as in GLM1. Several analyses were conducted with the parameters estimated from GLM2.

First, we searched for brain regions responding to prosocial decisions regardless of the audience effect in the positively valued organization. Two one-sample *t* tests were performed: (a) the trials in which the prosocial option (i.e., accept) was chosen were compared to those in which the selfish option (i.e., reject) was chosen (Fig 4B left, S4A Table); (b) then, we searched for brain regions in which brain activity for the accepted trials was modulated by the organization gain (S4B Table).

Second, because little is known about the brain networks engaged when being observed (i.e., public condition) or when making decisions in private regardless of the choice made, we performed 2 contrasts to test for the main effects of audience and privacy: public > private, and private > public, regardless of the organization types or of subjects’ choices (Fig 5, S7 Table).

To illustrate cerebral activity in the main regions revealed by these analyses, PSCs were extracted and averaged over subjects to create illustrative bar graphs of the contribution of each condition to the BOLD signal.

### Single-features analysis

A third model (GLM3) was used to investigate the brain regions representing single features, i.e., the moral cost and monetary benefit of the bad organization and the monetary cost and moral benefit of the charity. This model was similar to GLM1 except for the DV regressor, which was replaced by 2 orthogonal parametric regressors: (1) the amount of the moral cost/benefit (i.e., the size of the potential gain for the organization) and (2) the amount of the monetary cost versus benefit (i.e., the size of the potential gain or loss for the subject). For each of these regressors, the subject-specific parameter estimates were then entered in a within-subject factorial with 2 organization (positively versus negatively evaluated) × 2 observability (private versus public) conditions, allowing us to test for the following effects: (1) brain activity negatively modulated by the moral cost (i.e., organization gain in the negatively evaluated organization condition), (2) brain activity positively modulated by the monetary benefit (i.e., subject potential gain in the negatively evaluated organization), (3) brain activity positively modulated by moral benefit (i.e., organization gain in the positively evaluated organization), and (4) brain activity negatively modulated by monetary cost (i.e., subject potential loss in the positively evaluated organization) (S3 Fig and S4 Fig, S6 Table).

### Functional connectivity with DV-related brain regions

We used the CONN toolbox [94] (http://www.nitrc.org/projects/conn) to investigate whether the DV signals computed in the bilateral anterior insula for the bad organization—and in the ventral putamen for the charity condition—are passed to other brain regions. These functional connectivity analyses were performed taking the bilateral ventral putamen for the charity and bilateral anterior insula for the bad organization as seed regions, using 4 mm radius spheres centered on the peak voxels from the two DV signals (see S7 Table, S8 Table and S5 Fig, S6 Fig).

### Activations localization and reported statistics

Anatomic labeling of activated regions was performed using the SPM Anatomy toolbox (http://www.fz-juelich.de/inb/inb-3//spm_anatomy_toolbox) and the probabilistic atlas of Hammers. Reported coordinates conform to the Montreal Neurological Institute (MNI) space. Regions are listed in the tables that survived voxel-based thresholding of *p* < 0.001 uncorrected, and whole-brain cluster-level of *p* < 0.05 family-wise error (FWE) rate correction, except for a priori brain regions based on the literature in which small volume correction (SVC) was used with *p* < 0.05 FWE voxel-wise (indicated by “*”). A priori ROIs were the ventral putamen, the vmPFC, and the bilateral insula because these regions have been typically identified in neuroimaging studies on valuation [40], as well as charitable donation and social influence [15, 21, 22, 24, 35]. The anterior insula is also implicated in aversive processes [43] and in coding the negative valence of subjective value [61], and the DLPFC is known for its engagement in moral rules as aversive outcomes [28] and in making decisions concerning dishonesty [29–31, 33]. The SVC was performed in 9-mm spheres centered on the coordinates of the peak activity revealed by a previous meta-analysis on the neural correlates of subjective value: in left and right ventral putamen (x, y, z = −12, 4, 2 and 12, 10, −2), in the vmPFC (x, y, z = 2, 46, −8), and in the left and right anterior insula (x, y, z = −30, 22, −6 and 32, 20, −6) [40]. The coordinates of the DLPFC ROI (x, y, z = −39, 37, 22) were based on a classical paper suggesting that different types of economic norm enforcement following immoral actions may be supported by common DLPFC regions [78].

## Supporting information

S1 Data (XLSX)

S1 Fig Color-coded heatmaps of probability of acceptance for each dilemma of the 8 × 8 monetary versus moral gain versus loss matrix. Red indicates high willingness to accept, and blue indicates low willingness to accept. One heatmap is drawn for each organization and each observation condition. See S1 Data.
(TIF)

S2 Fig Color-coded heatmaps of RTs for each dilemma in a 4 × 4 monetary versus moral gain versus loss matrix. Red indicates slower RTs, and blue indicates faster RTs. One heatmap is drawn for each organization and each observation condition. See S1 Data. RT, response time.
(TIF)

S3 Fig **In the negatively evaluated organization, whole-brain analysis of parametric responses to size of potential moral cost (left) or monetary gain to the subject (right).** Statistical maps were projected onto the ch2bet template of MRICroN software; coronal slices (y = 23) are included to show anterior insula activations. For display purposes, all maps are thresholded with a *p*-value of *p* < 0.005 uncorrected. See also S5 Table.
(TIF)

S4 Fig **In the positively evaluated organization (charity), whole-brain analysis of parametric responses to size of potential monetary cost (left) or moral benefit (right).** Statistical maps were projected onto the ch2bet template of MRICroN software; coronal slices (y = 25 and y = 50) show midbrain and vmPFC activation, respectively. For display purposes, maps are thresholded with a *p*-value of *p* < 0.005 uncorrected. See S5 Table. vmPFC, ventromedial prefrontal cortex.
(TIF)

S5 Fig Connectivity with the DV-related anterior insula in the negatively evaluated organization. Seed-to-voxel functional connectivity maps showing the strength of the correlation between seeds in the left (a) and right (b) anterior insula identified in the correlation with DV in the bad cause condition (x, y, z = : −36, 14, 1; x, y, z = 36, 14, 1), using the CONN toolbox [94]. Cluster FDR-corrected *p* < 0.001. Positive correlations are shown in red and negative correlations in blue. DV, decision value; FDR, false discovery rate.
(TIF)

S6 Fig Connectivity with the DV-related ventral striatum in the positively evaluated organization. Seed-to-voxel functional connectivity maps showing the strength of the correlation between seeds in the left (a) and right (b) ventral striatum identified in the correlation with DV in the charity condition (x, y, z = : -21, 14, −2; x, y, z = 15, 17, −2), using the CONN toolbox [94]. Cluster FDR-corrected *p* < 0.001. Positive correlations are shown in red and negative in blue. DV, decision value; FDR, false discovery rate.
(PPTX)

S1 Table Comparisons of the model fit (AIC and BIC) of probit and logistic models of model 1 (first 4 lines) and model 4 (lines 5 to 8). AIC,; BIC,.
(DOCX)

S2 Table Related to behavioral results and Figs 2, 3 and 4. Results of the random effect logistic regression analyses for the behavioral models 1 to 4.
(DOCX)

S3 Table Related to Fig 3A and Fig 4A. **Brain areas whose activity significantly correlated with DV for the negatively and the positively evaluated organizations (MNI coordinates and statistic *t*).** DV, decision value; MNI, Montreal Neurological Institute.
(DOCX)

S4 Table Related to Fig 4B. **In the positively evaluated organization, brain regions engaged with selection of the prosocial option, and brain regions showing increasing activity with higher moral benefit.** (a) Brain region whose activity was engaged with selection of the prosocial option (accept > reject) for the charity. (b) Brain region whose activity increased with higher moral benefit for accepted trials only in the charity condition (MNI coordinates and statistic *t*). MNI, Montreal Neurological Institute.
(DOCX)

S5 Table Related to Fig 5. MNI coordinates and statistic *t* for the main effect of audience (public > private) and the main effect of privacy (private > public), regardless of choices.
(DOCX)

S6 Table Related to S3 Fig and S4 Fig. **Brain regions showing correlations with single attributes, i.e., monetary cost, monetary benefit, moral cost, and moral benefit (MNI coordinates and statistic *t*).** MNI, Montreal Neurological Institute.
(DOCX)

S7 Table Related to S5 Fig. **Functionally interconnected brain regions (seed to voxel) with DV-related anterior insula in the negatively evaluated organization.** Seed anterior insula Left ROI (a) is a 4 mm radius sphere with coordinates x, y, z = −36, 14, −1, and seed anterior insula Right ROI is a 4 mm sphere with coordinates x, y, z = 36, 20, 1. MNI coordinates of peak. DV, decision value; MNI, Montreal Neurological Institute; ROI, region of interest.
(DOCX)

S8 Table Related to S6 Fig. **Functionally interconnected brain regions (seed to voxel) with the DV-related ventral putamen in the positively evaluated organization.** Seed ventral putamen Left ROI (a) is a 4 mm radius sphere with coordinates x, y, z = −21, 14, −2, and seed ventral putamen Right ROI is a 4 mm sphere with coordinates x, y, z = 15, 17, −2. MNI coordinates of peak. DV, decision value; MNI, Montreal Neurological Institute; ROI, region of interest.
(DOCX)

## Abbreviations

ACC: anterior cingulate cortex
BOLD: blood-oxygenation-level–dependent
dACC: dorsal ACC
DLPFC: dorsolateral prefrontal cortex
DV: decision value
EEG: electroencephalography
EPI: echo-planar image
fMRI: functional MRI
FWE: family-wise error
FWHM: full width at half maximum
GLM: general linear model
HRF: hemodynamic response function
ITI: inter-trial interval
MNI: Montreal Neurological Institute
PFC: prefrontal cortex
PSC: percent signal change
ROI: region of interest
RT: response time
SVC: small volume correction
TMS: transcranial magnetic stimulation
TPJ: temporoparietal junction
vmPFC: ventromedial prefrontal cortex

## References

[1] Allingham MG, Sandmo A. Income tax evasion: A theoretical analysis. Journal of Public Economics. 1972;1(3–4):323–38.

[2] Becker GS. Crime and punishment: An economic approach The Economic Dimensions of Crime: Springer; 1968 p. 13–68.

[3] Hechter M. The attainment of solidarity in intentional communities. Rationality and Society. 1990;2(2):142–55.

[4] Lewicki RJ. Lying and deception: A behavioral model. Negotiating in Organizations. 1983;68:90.

[5] Aronson E. The theory of cognitive dissonance: A current perspective. Advances in Experimental Social Psychology. 1969;4:1–34.

[6] Harris S, Mussen P, Rutherford E. Maturity of moral judgment. The Journal of Genetic Psychology. 1976;128(1):123–35. 1255137 10.1080/00221325.1976.10533980

[7] Fischbacher U, Föllmi‐Heusi F. Lies in disguise—an experimental study on cheating. Journal of the European Economic Association. 2013;11(3):525–47.

[8] Irlenbusch B, Villeval MC. Behavioral ethics: how psychology influenced economics and how economics might inform psychology? Current Opinion in Psychology. 2015;6:87–92.

[9] Mazar N, Amir O, Ariely D. The dishonesty of honest people: A theory of self-concept maintenance. Journal of Marketing Research. 2008;45(6):633–44.

[10] Shalvi S, Dana J, Handgraaf MJ, De Dreu CK. Justified ethicality: Observing desired counterfactuals modifies ethical perceptions and behavior. Organizational Behavior and Human Decision Processes. 2011;115(2):181–90.

[11] Gneezy U, Kajackaite A, Sobel J. Lying Aversion and the Size of the Lie. American Economic Review. 2018;108(2):419–53.

[12] Kajackaite A, Gneezy U. Incentives and cheating. Games and Economic Behavior. 2017;102:433–44.

[13] Ariely D, Bracha A, Meier S. Doing Good or Doing Well? Image Motivation and Monetary Incentives in Behaving Prosocially. American Economic Review. 2009;99(1):544–55. 10.1257/aer.99.1.544 WOS:000264785500022.

[14] Bohnet I, Frey BS. The sound of silence in prisoner's dilemma and dictator games. Journal of Economic Behavior & Organization. 1999;38(1):43–57.

[15] Izuma K, Saito DN, Sadato N. Processing of social and monetary rewards in the human striatum. Neuron. 2008;58(2):284–94. 10.1016/j.neuron.2008.03.020 18439412

[16] Bénabou R, Tirole J. Incentives and prosocial behavior. American Economic Review. 2006;96(5):1652–78. 10.1257/aer.96.5.1652 WOS:000243238500011.

[17] Kable JW, Glimcher P. The neural correlates of subjective value during intertemporal choice. Nature Neuroscience. 2007;10(12):1625 10.1038/nn2007 17982449 PMC2845395

[18] Prévost C, Pessiglione M, Météreau E, Cléry-Melin M-L, Dreher J-C. Separate valuation subsystems for delay and effort decision costs. Journal of Neuroscience. 2010;30(42):14080–90. 10.1523/JNEUROSCI.2752-10.2010 20962229 PMC6634773

[19] Rangel A, Hare TA. Neural computations associated with goal-directed choice. Current Opinion in Neurobiology. 2010;20(2):262–70. 10.1016/j.conb.2010.03.001 20338744

[20] Cooper JC, Kreps TA, Wiebe T, Pirkl T, Knutson B. When giving is good: ventromedial prefrontal cortex activation for others' intentions. Neuron. 2010;67(3):511–21. 10.1016/j.neuron.2010.06.030 20696386 PMC2919841

[21] Harbaugh WT, Mayr U, Burghart DR. Neural responses to taxation and voluntary giving reveal motives for charitable donations. Science. 2007;316(5831):1622–5. 10.1126/science.1140738 17569866

[22] Hare TA, Camerer CF, Knoepfle DT, O'Doherty JP, Rangel A. Value computations in ventral medial prefrontal cortex during charitable decision making incorporate input from regions involved in social cognition. Journal of Neuroscience. 2010;30(2):583–90. 10.1523/JNEUROSCI.4089-09.2010 20071521 PMC6633003

[23] Hutcherson CA, Bushong B, Rangel A. A neurocomputational model of altruistic choice and its implications. Neuron. 2015;87(2):451–62. 10.1016/j.neuron.2015.06.031 26182424 PMC4947370

[24] Moll J, Krueger F, Zahn R, Pardini M, de Oliveira-Souza R, Grafman J. Human fronto–mesolimbic networks guide decisions about charitable donation. Proceedings of the National Academy of Sciences. 2006;103(42):15623–8. 10.1073/pnas.0604475103 PMC1622872 17030808

[25] Tricomi E, Rangel A, Camerer CF, O’Doherty JP. Neural evidence for inequality-averse social preferences. Nature. 2010;463(7284):1089–91. 10.1038/nature08785 20182511

[26] Zaki J, Mitchell JP. Equitable decision making is associated with neural markers of intrinsic value. Proceedings of the National Academy of Sciences. 2011;108(49):19761–6. 10.1073/pnas.1112324108 PMC3241792 22106300

[27] Rangel A, Camerer C, Montague PR. A framework for studying the neurobiology of value-based decision making. Nature Reviews Neuroscience. 2008;9(7):545 10.1038/nrn2357 18545266 PMC4332708

[28] Xiang T, Lohrenz T, Montague PR. Computational substrates of norms and their violations during social exchange. Journal of Neuroscience. 2013;33(3):1099–108. 10.1523/JNEUROSCI.1642-12.2013 23325247 PMC3631781

[29] Dogan A, Morishima Y, Heise F, Tanner C, Gibson R, Wagner AF, et al Prefrontal connections express individual differences in intrinsic resistance to trading off honesty values against economic benefits. Scientific Reports. 2016;6:33263 10.1038/srep33263 27646044 PMC5028845

[30] Greene JD, Paxton JM. Patterns of neural activity associated with honest and dishonest moral decisions. Proceedings of the National Academy of Sciences. 2009;106(30):12506–11. 10.1073/pnas.0900152106 PMC2718383 19622733

[31] Zhu L, Jenkins AC, Set E, Scabini D, Knight RT, Chiu PH, et al Damage to dorsolateral prefrontal cortex affects tradeoffs between honesty and self-interest. Nature Neuroscience. 2014;17(10):1319 10.1038/nn.3798 25174003 PMC4177007

[32] Baumgartner T, Knoch D, Hotz P, Eisenegger C, Fehr E. Dorsolateral and ventromedial prefrontal cortex orchestrate normative choice. Nature Neuroscience. 2011;14(11):1468 10.1038/nn.2933 21964488

[33] Buckholtz JW, Asplund CL, Dux PE, Zald DH, Gore JC, Jones OD, et al The Neural Correlates Of Third-Party Punishment. Neuron. 2008;60(5):930–40. 10.1016/j.neuron.2008.10.016 19081385

[34] Ruff CC, Ugazio G, Fehr E. Changing social norm compliance with noninvasive brain stimulation. Science. 2013;342(6157):482–4. 10.1126/science.1241399 24091703

[35] Spitzer M, Fischbacher U, Herrnberger B, Grön G, Fehr E. The neural signature of social norm compliance. Neuron. 2007;56(1):185–96. 10.1016/j.neuron.2007.09.011 17920024

[36] Saxe R, Kanwisher N. People thinking about thinking people: the role of the temporo-parietal junction in “theory of mind”. Neuroimage. 2003;19(4):1835–42. 12948738 10.1016/s1053-8119(03)00230-1

[37] Hare TA, O'Doherty J, Camerer CF, Schultz W, Rangel A. Dissociating the role of the orbitofrontal cortex and the striatum in the computation of goal values and prediction errors. Journal of Neuroscience. 2008;28(22):5623–30. 10.1523/JNEUROSCI.1309-08.2008 18509023 PMC6670807

[38] Amodio DM, Frith CD. Meeting of minds: the medial frontal cortex and social cognition. Nature Reviews Neuroscience. 2006;7(4):268–77. 10.1038/nrn1884 . 16552413

[39] Meyer ML, Spunt RP, Berkman ET, Taylor SE, Lieberman M. Evidence for social working memory from a parametric functional MRI study. Proceedings of the National Academy of Sciences. 2012;109(6):1883–8. 10.1073/pnas.1121077109 PMC3277536 22308468

[40] Bartra O, McGuire JT, Kable JW. The valuation system: a coordinate-based meta-analysis of BOLD fMRI experiments examining neural correlates of subjective value. Neuroimage. 2013;76:412–27. 10.1016/j.neuroimage.2013.02.063 23507394 PMC3756836

[41] Fouragnan E, Retzler C, Mullinger K, Philiastides M. Two spatiotemporally distinct value systems shape reward-based learning in the human brain. Nature Communications. 2015;6:8107 10.1038/ncomms9107 26348160 PMC4569710

[42] Haber SN, Knutson B. The reward circuit: linking primate anatomy and human imaging. Neuropsychopharmacology. 2010;35(1):4 10.1038/npp.2009.129 19812543 PMC3055449

[43] Liu X, Hairston J, Schrier M, Fan J. Common and distinct networks underlying reward valence and processing stages: a meta-analysis of functional neuroimaging studies. Neuroscience & Biobehavioral Reviews. 2011;35(5):1219–36. 21185861 10.1016/j.neubiorev.2010.12.012 PMC3395003

[44] Eisenberger NI, Lieberman MD, Williams KD. Does rejection hurt? An fMRI study of social exclusion. Science. 2003;302(5643):290–2. 10.1126/science.1089134 14551436

[45] Sanfey AG, Rilling JK, Aronson JA, Nystrom LE, Cohen JD. The neural basis of economic decision-making in the ultimatum game. Science. 2003;300(5626):1755–8. 10.1126/science.1082976 12805551

[46] Crockett MJ, Siegel JZ, Kurth-Nelson Z, Dayan P, Dolan R. Moral transgressions corrupt neural representations of value. Nature Neuroscience. 2017;20(6):879 10.1038/nn.4557 28459442 PMC5462090

[47] Rilling JK, Gutman DA, Zeh TR, Pagnoni G, Berns GS, Kilts CD. A neural basis for social cooperation. Neuron. 2002;35(2):395–405. 12160756 10.1016/s0896-6273(02)00755-9

[48] Andreoni J. Impure altruism and donations to public goods: A theory of warm-glow giving. The Economic Journal. 1990;100(401):464–77.

[49] Lopez-Persem A, Domenech P, Pessiglione M. How prior preferences determine decision-making frames and biases in the human brain. Elife. 2016 11 19;5 pii: e20317. 10.7554/eLife.20317 27864918 PMC5132340

[50] Allen JD, Davison CS. Effects of caudate lesions on signaled and nonsignaled sidman avoidance in the rat. Behavioral Biology. 1973;8(2):239–50. 4705973 10.1016/s0091-6773(73)80023-9

[51] Li M, Davidson P, Budin R, Kapur S, Fleming ASJSR. Effects of typical and atypical antipsychotic drugs on maternal behavior in postpartum female rats. Schizophrenia Research. 2004;70(1):69–80. 10.1016/j.schres.2003.09.013 15246466

[52] McCullough L, Sokolowski J, Salamone JJN. A neurochemical and behavioral investigation of the involvement of nucleus accumbens dopamine in instrumental avoidance. Neuroscience. 1993;52(4):919–25. 8450978 10.1016/0306-4522(93)90538-q

[53] Neill DB, Ross JF, Grossman SPJPB, Behavior. Effects of lesions in the dorsal or ventral striatum on locomotor activity and on locomotor effects of amphetamine. Pharmacology Biochemistry and Behavior. 1974;2(5):697–702. 10.1016/0091-3057(74)90040-9 4610596

[54] Winocur G, Mills JAJJoC, Psychology P. Effects of caudate lesions on avoidance behavior in rats. Journal of Comparative and Physiological Psychology. 1969;68(4):552 5344505 10.1037/h0027645

[55] Delgado MR, Jou RL, Ledoux JE, Phelps EA. (2009) Avoiding negative outcomes: tracking the mechanisms of avoidance learning in humans during fear conditioning. Front Behav Neurosci 3:33 10.3389/neuro.08.033.2009 19847311 PMC2762377

[56] Seymour B, Daw ND, Roiser JP, Dayan P, Dolan R. (2012) Serotonin selectively modulates reward value in human decision-making. J Neurosci 32:5833–5842. 10.1523/JNEUROSCI.0053-12.2012 22539845 PMC5321452

[57] Eldar E, Hauser TU, Dayan P, Dolan RJ. (2016) Striatal structure and function predict individual biases in learning to avoid pain. Proc Natl Acad Sci U S A 113:4812–4817. 10.1073/pnas.1519829113 27071092 PMC4855606

[58] Schlund MW, Brewer AT, Magee SK, Richman DM, Solomon S, Ludlum M, Dymond S. (2016) The tipping point: value differences and parallel dorsal-ventral frontal circuits gating human approach-avoidance behavior. Neuroimage 136:94–105. 10.1016/j.neuroimage.2016.04.070 27153979

[59] Boeke Moscarello, LeDoux Phelps, Hartley. Active Avoidance: Neural Mechanisms and Attenuation of Pavlovian Conditioned Responding. J Neurosci. 2017 5 3; 37(18): 4808–4818. 10.1523/JNEUROSCI.3261-16.2017 28408411 PMC5426570

[60] Rudebeck PH, Walton ME, Smyth AN, Bannerman DM, Rushworth MF. Separate neural pathways process different decision costs. Nature Neuroscience. 2006;9(9):1161–8. 10.1038/nn1756 16921368

[61] Ashar YK, Andrews-Hanna JR, Dimidjian S, Wager TD. Empathic care and distress: predictive brain markers and dissociable brain systems. Neuron. 2017;94(6):1263–73. e4. 10.1016/j.neuron.2017.05.014 28602689 PMC5532453

[62] Zaki J, Ochsner K. The neuroscience of empathy: progress, pitfalls and promise. Nature Neuroscience. 2012;15(5):675 10.1038/nn.3085 22504346

[63] Kahnt T, Park SQ, Haynes JD, Tobler PN. Disentangling neural representations of value and salience in the human brain. Proceedings of the National Academy of Sciences. 2014; 201320189 10.1073/pnas.1320189111 PMC3977254 24639493

[64] Météreau E, Dreher J-C. Cerebral correlates of salient prediction error for different rewards and punishments. Cerebral Cortex. 2012;23(2):477–87. 10.1093/cercor/bhs037 22368086

[65] Waytz A, Zaki J, Mitchell J. Response of dorsomedial prefrontal cortex predicts altruistic behavior. Journal of Neuroscience. 2012;32(22):7646–50. 10.1523/JNEUROSCI.6193-11.2012 22649243 PMC3387686

[66] Delgado MR, Beer JS, Fellows LK, Huettel SA, Platt ML, Quirk GJ, et al Viewpoints: Dialogues on the functional role of the ventromedial prefrontal cortex. Nature Neuroscience. 2016;19(12):1545 10.1038/nn.4438 27898086

[67] Kolling N, Behrens TE, Mars RB, Rushworth MF. Neural mechanisms of foraging. Science. 2012;336(6077):95–8. 10.1126/science.1216930 22491854 PMC3440844

[68] Shenhav A, Buckner RL. Neural correlates of dueling affective reactions to win–win choices. Proceedings of the National Academy of Sciences. 2014;111(30):10978–83. 10.1073/pnas.1405725111 PMC4121780 25024178

[69] Neubert F-X, Mars RB, Sallet J, Rushworth MF. Connectivity reveals relationship of brain areas for reward-guided learning and decision making in human and monkey frontal cortex. Proceedings of the National Academy of Sciences. 2015:201410767. 10.1073/pnas.1410767112 PMC4443352 25947150

[70] Klucharev V, Hytonen K, Rijpkema M, Smidts A, Fernandez G. Reinforcement learning signal predicts social conformity. Neuron. 2009;61(1):140–151. 10.1016/j.neuron.2008.11.027 19146819

[71] Park SQ, Kahnt T, Rieskamp J, Heekeren HR. Neurobiology of value integration: when value impacts valuation. Journal of Neuroscience. 2011;31(25):9307–14. 10.1523/JNEUROSCI.4973-10.2011 21697380 PMC6623498

[72] Wu H, Luo Y, Feng C. Neural signatures of social conformity: A coordinate-based activation likelihood estimation meta-analysis of functional brain imaging studies. Neuroscience Biobehavior Review. 2016;71:101–11. 10.1016/j.neubiorev.2016.08.038 27592151

[73] Zaki J, Schirmer J, Mitchell J. Social influence modulates the neural computation of value. Psychological Science. 2011;22(7):894–900. 10.1177/0956797611411057 21653908

[74] Obeso I, Moisa M, Ruff CC, Dreher J-C. A causal role for right temporo-parietal junction in signaling moral conflict. eLife. 2018;7:e40671 10.7554/eLife.40671 30561334 PMC6298767

[75] Ugazio G, Ruff C. Neural Control of Social Decisions: Causal Evidence From Brain Stimulation Studies. Decision Neuroscience. 2017;p. 233–45.

[76] Saxe R, Powell LJ. It's the thought that counts: specific brain regions for one component of theory of mind. Psychological Science. 2006;17(8):692–9. 10.1111/j.1467-9280.2006.01768.x 16913952

[77] Greene JD, Nystrom LE, Engell AD, Darley JM, Cohen JD. The neural bases of cognitive conflict and control in moral judgment. Neuron. 2004;44(2):389–400. 10.1016/j.neuron.2004.09.027 15473975

[78] Buckholtz JW, Martin JW, Treadway MT, Jan K, Zald DH, Jones O, et al From blame to punishment: disrupting prefrontal cortex activity reveals norm enforcement mechanisms. Neuron. 2015;87(6):1369–80. 10.1016/j.neuron.2015.08.023 26386518 PMC5488876

[79] FeldmanHall O, Dalgleish T, Evans D, Mobbs D. Empathic concern drives costly altruism. Neuroimage. 2015;105:347–56. 10.1016/j.neuroimage.2014.10.043 25462694 PMC4275572

[80] Berns GS, Bell E, Capra CM, Prietula MJ, Moore S, Anderson B, et al The price of your soul: neural evidence for the non-utilitarian representation of sacred values. Social Science Electronic Publishing. 2012;367(1589):754–62. 10.1098/rstb.2011.0262 PMC3260841 22271790

[81] FeldmanHall O, Dalgleish T, Thompson R, Evans D, Schweizer S, Mobbs D. Differential neural circuitry and self-interest in real vs hypothetical moral decisions. Social Cognitive Affective Neuroscience. 2012;7(7):743–51. 10.1093/scan/nss069 22711879 PMC3475363

[82] Andreoni J, Vesterlund L. Which is the fair sex? Gender differences in altruism. The Quarterly Journal of Economics. 2001;116(1):293–312.

[83] Eckel CC, Grossman PJ. Are women less selfish than men?: Evidence from dictator experiments. The Economic Journal. 1998;108(448):726–35.

[84] Childs J. Gender differences in lying. Economics Letters. 2012;114(2):147–9.

[85] Dreber A, Johannesson M. Gender differences in deception. Economics Letters. 2008;99(1):197–9.

[86] Dreher J-C, Schmidt PJ, Kohn P, Furman D, Rubinow D, Berman KF. Menstrual cycle phase modulates reward-related neural function in women. Proceedings of the National Academy of Sciences. 2007;104(7):2465–70. 10.1073/pnas.0605569104 PMC1892961 17267613

[87] Wheeler SC, Petty RE. The effects of stereotype activation on behavior: a review of possible mechanisms. Psychological Bulletin. 2001;127(6):797 11726072 10.1037/0033-2909.127.6.797

[88] Spencer SJ, Steele CM, Quinn DM. Stereotype threat and women's math performance. Journal of Experimental Social Psychology. 1999;35(1):4–28.

[89] Abeler J, Becker A, Falk A. Representative Evidence on Lying Costs. Journal of Public Economics. 2014;113:96–104.

[90] Hallsworth M, Berry D, Sanders M, Sallis A, King D, Vlaev I, et al Stating appointment costs in SMS reminders reduces missed hospital appointments: findings from two randomised controlled trials. PLoS ONE. 2015;10(9):e0137306 10.1371/journal.pone.0137306 26366885 PMC4569397

[91] Ito K, Ida T, Tanaka M. The Persistence of Moral Suasion and Economic Incentives: Field Experimental Evidence from Energy Demand. American Economic Journal: Economic Policy. 2018;10(1):240–67.

[92] Mazaika P, Hoeft F, Glover G, Reiss A. Methods and software for fMRI analysis for clinical subjects (poster presented at Stanford University Center for Interdisciplinary Brain Sciences Research). 2009; Stanford University, Stanford, California.

[93] Peters J, Büchel C. Neural representations of subjective reward value. Behavioural brain research. 2010;213(2):135–41. 10.1016/j.bbr.2010.04.031 20420859

[94] Whitfield-Gabrieli S, Nieto-Castanon A. Conn: a functional connectivity toolbox for correlated and anticorrelated brain networks. Brain Connectivity. 2012;2(3):125–41. 10.1089/brain.2012.0073 . 22642651

