# Supplementary figures and images for: Neurocomputational mechanisms at play when weighing concerns for extrinsic rewards, moral values, and social image
Source: PLoS Biol. 2019 Jun 6;17(6):e3000283. doi: 10.1371/journal.pbio.3000283 (PMC6553686; doi:10.1371/journal.pbio.3000283)

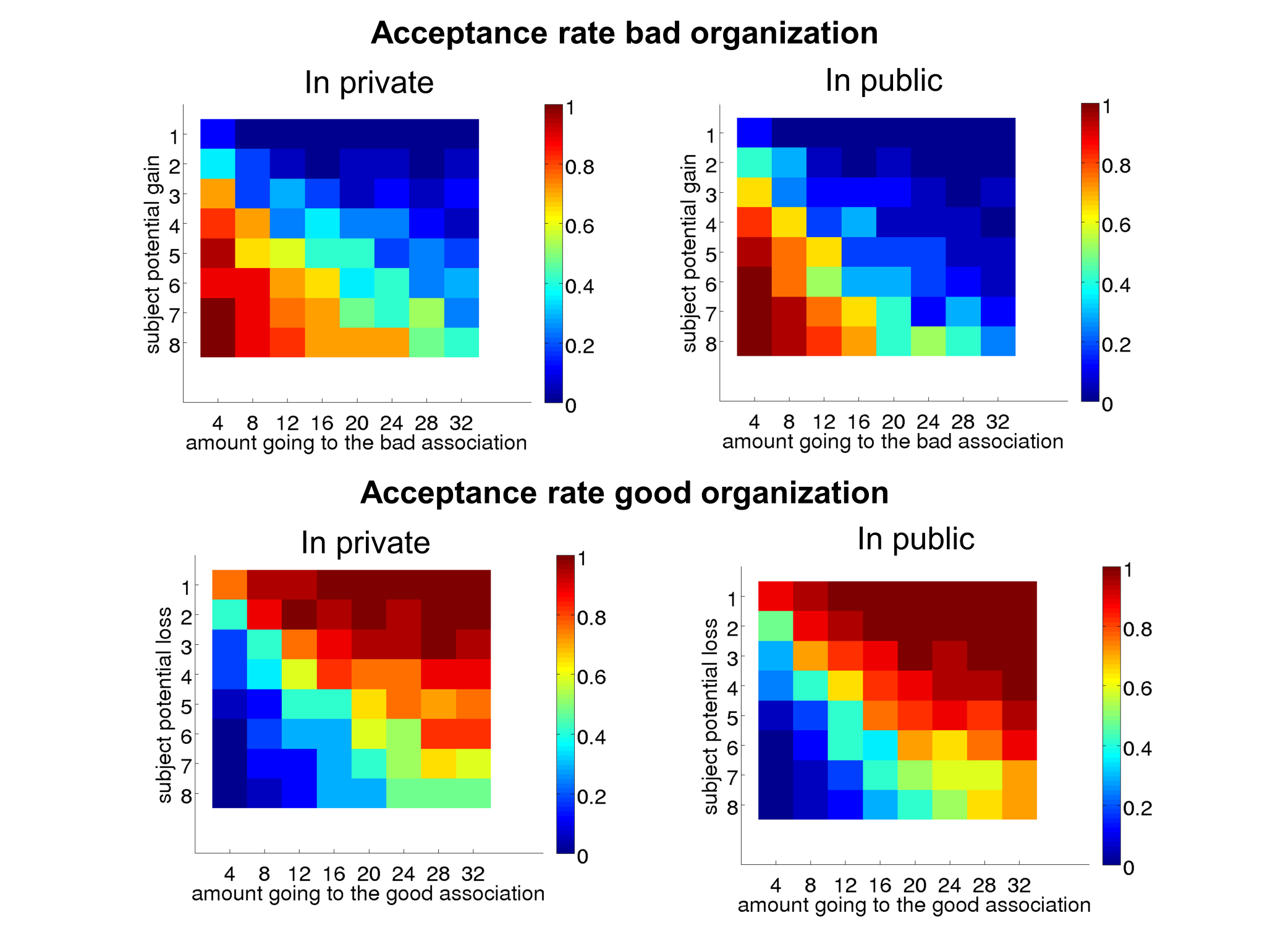

Supplement: S1 Fig — Red indicates high willingness to accept, and blue indicates low willingness to accept. One heatmap is drawn for each organization and each observation condition. See S1 Data. (TIF) [file pbio.3000283.s002.tif]

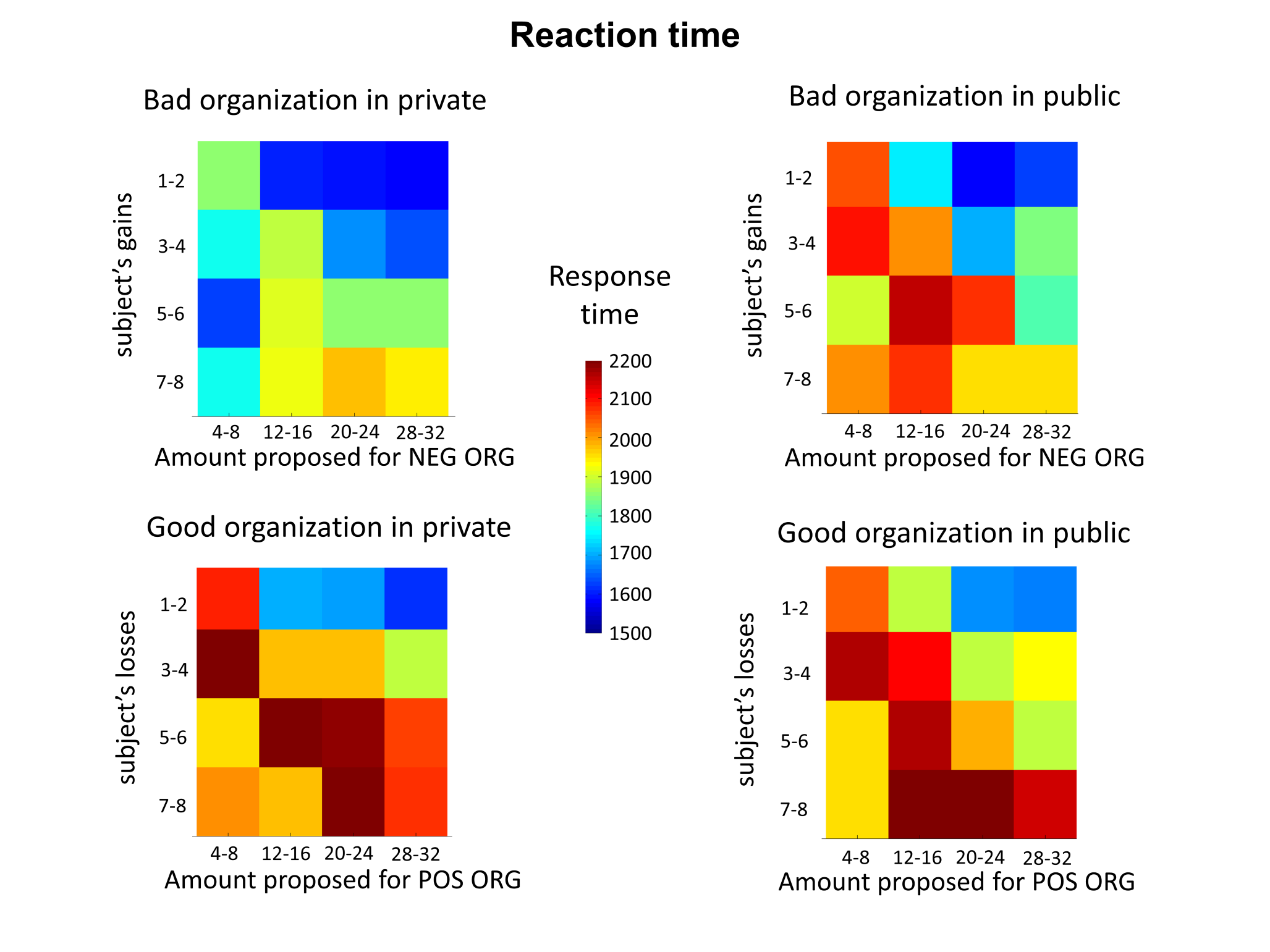

Supplement: S2 Fig — Red indicates slower RTs, and blue indicates faster RTs. One heatmap is drawn for each organization and each observation condition. See S1 Data. RT, response time. (TIF) [file pbio.3000283.s003.tif]

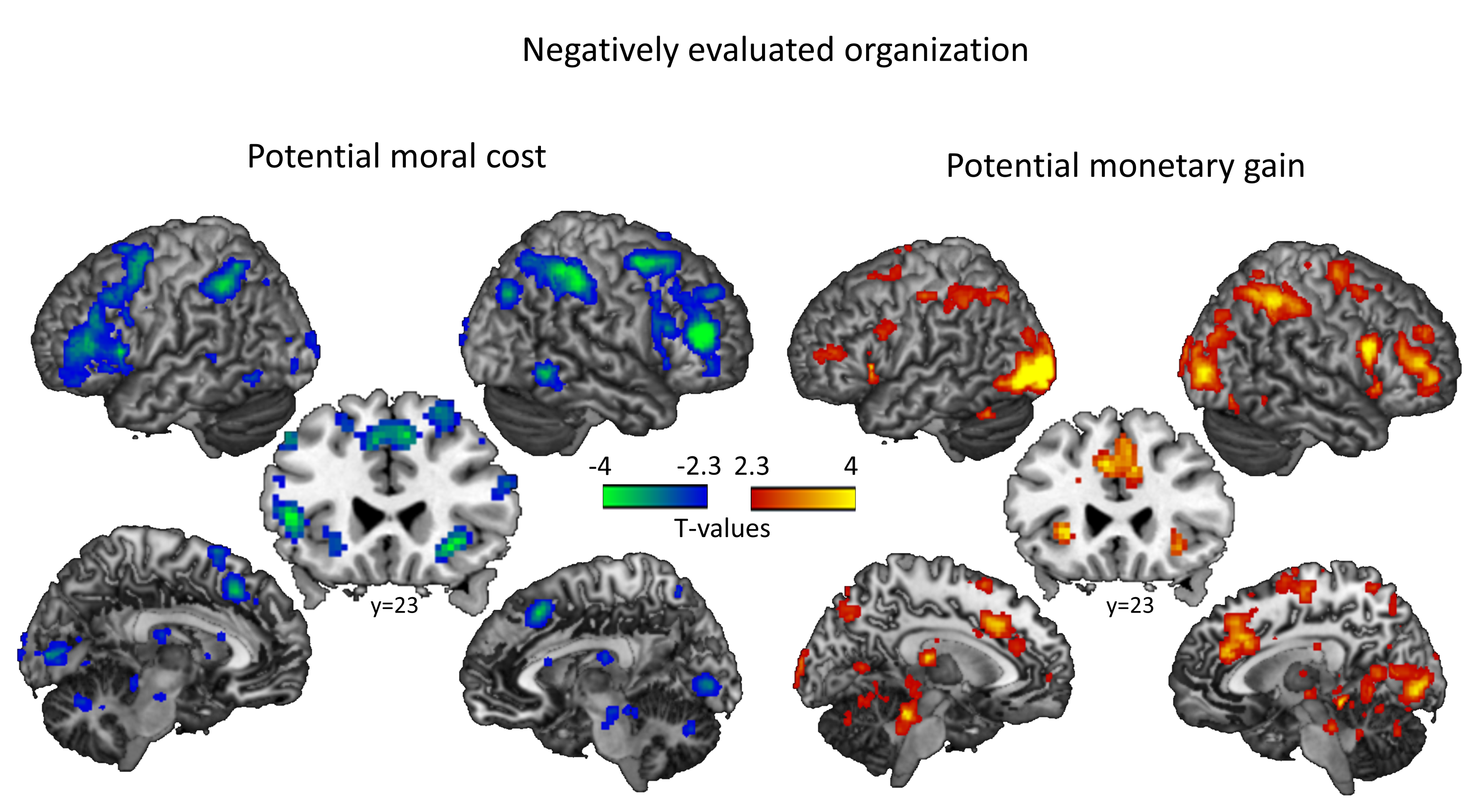

Supplement: S3 Fig — In the negatively evaluated organization, whole-brain analysis of parametric responses to size of potential moral cost (left) or monetary gain to the subject (right). Statistical maps were projected onto the ch2bet template of MRICroN software; coronal slices (y = 23) are included to show anterior insula activations. For display purposes, all maps are thresholded with a p-value of p < 0.005 uncorrected. See also S5 Table. (TIF) [file pbio.3000283.s004.tif]

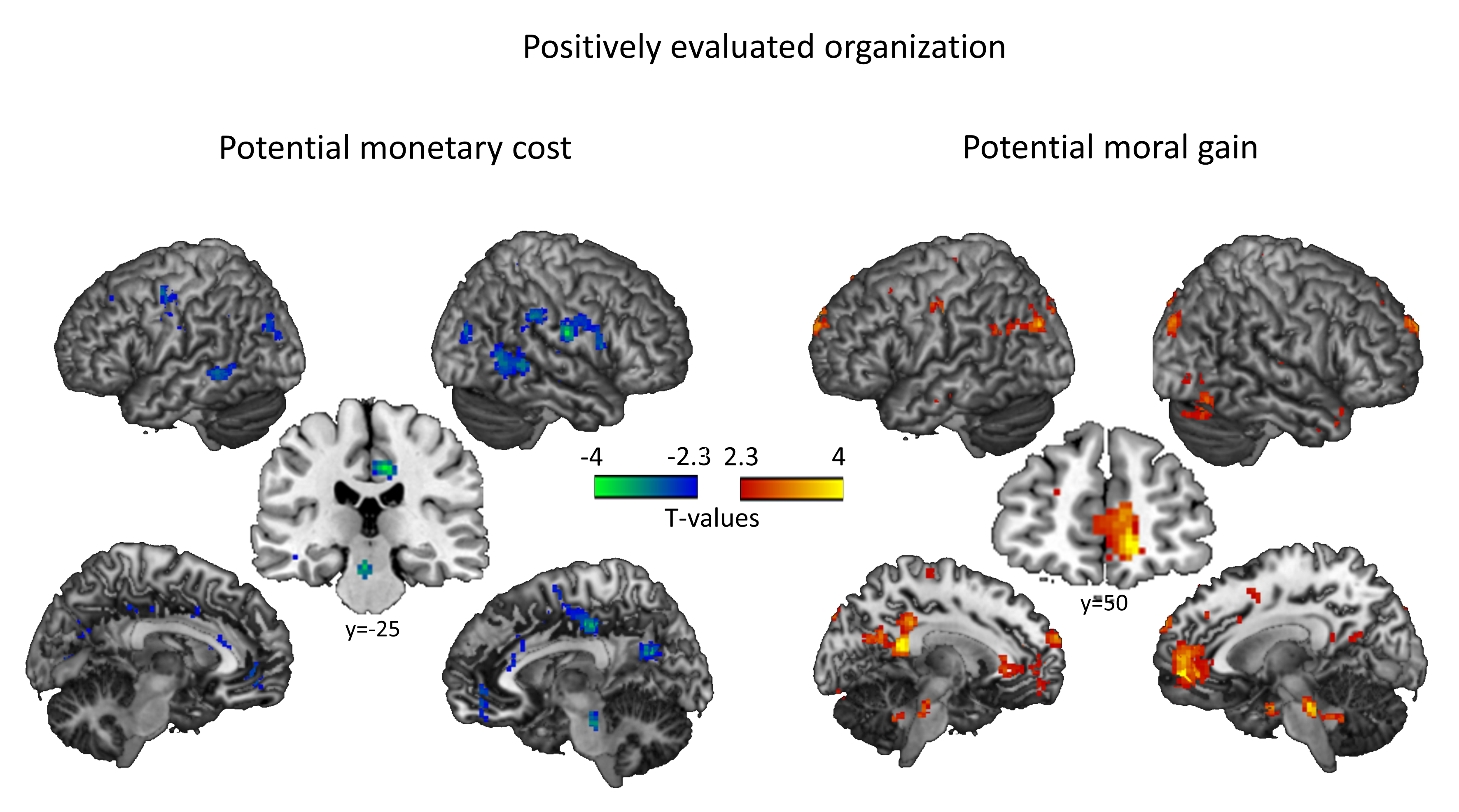

Supplement: S4 Fig — In the positively evaluated organization (charity), whole-brain analysis of parametric responses to size of potential monetary cost (left) or moral benefit (right). Statistical maps were projected onto the ch2bet template of MRICroN software; coronal slices (y = 25 and y = 50) show midbrain and vmPFC activation, respectively. For display purposes, maps are thresholded with a p-value of p < 0.005 uncorrected. See S5 Table. vmPFC, ventromedial prefrontal cortex. (TIF) [file pbio.3000283.s005.tif]

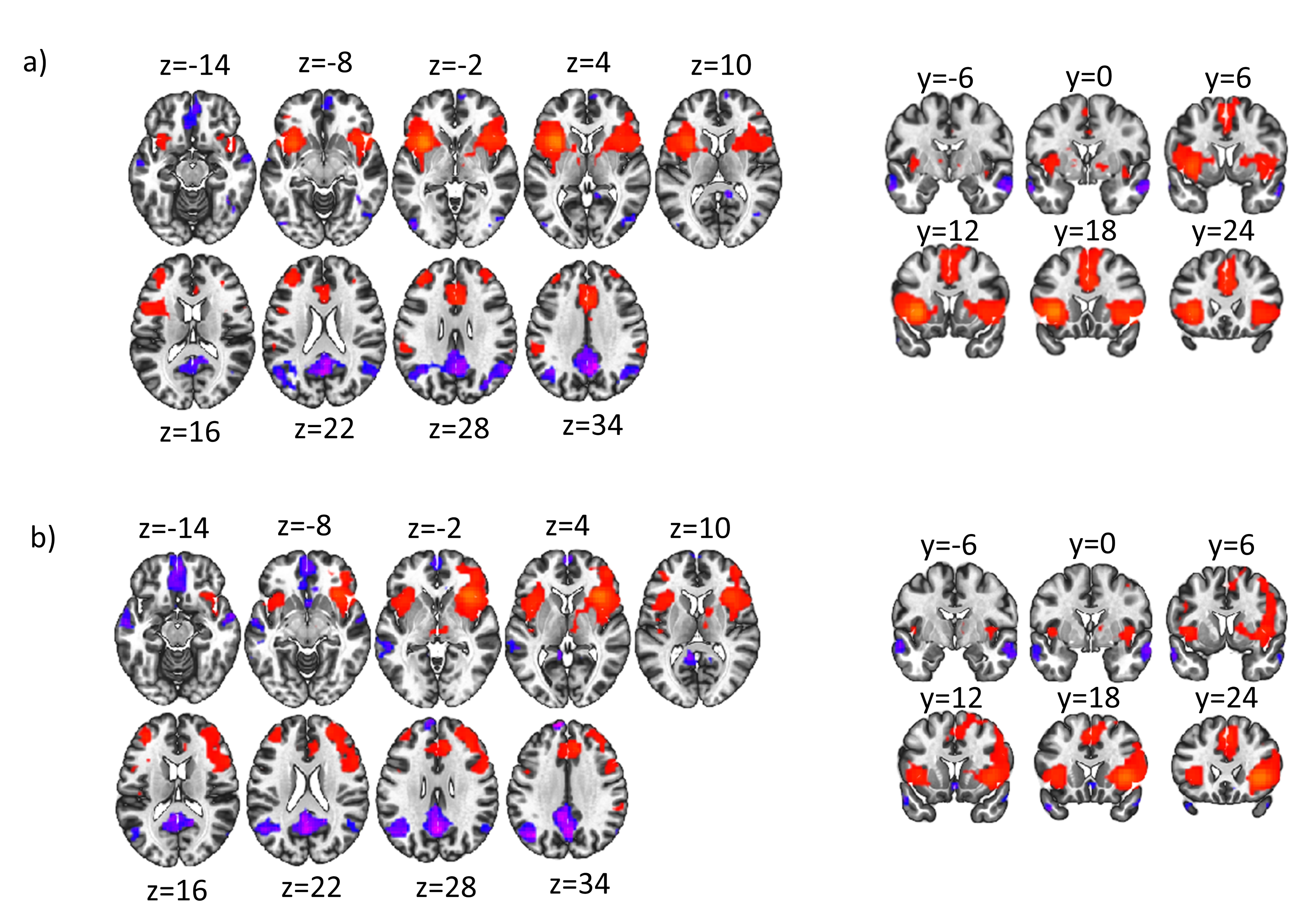

Supplement: S5 Fig — Seed-to-voxel functional connectivity maps showing the strength of the correlation between seeds in the left (a) and right (b) anterior insula identified in the correlation with DV in the bad cause condition (x, y, z = : −36, 14, 1; x, y, z = 36, 14, 1), using the CONN toolbox [94]. Cluster FDR-corrected p < 0.001. Positive correlations are shown in red and negative correlations in blue. DV, decision value; FDR, false discovery rate. (TIF) [file pbio.3000283.s006.tif]

## Slide 1
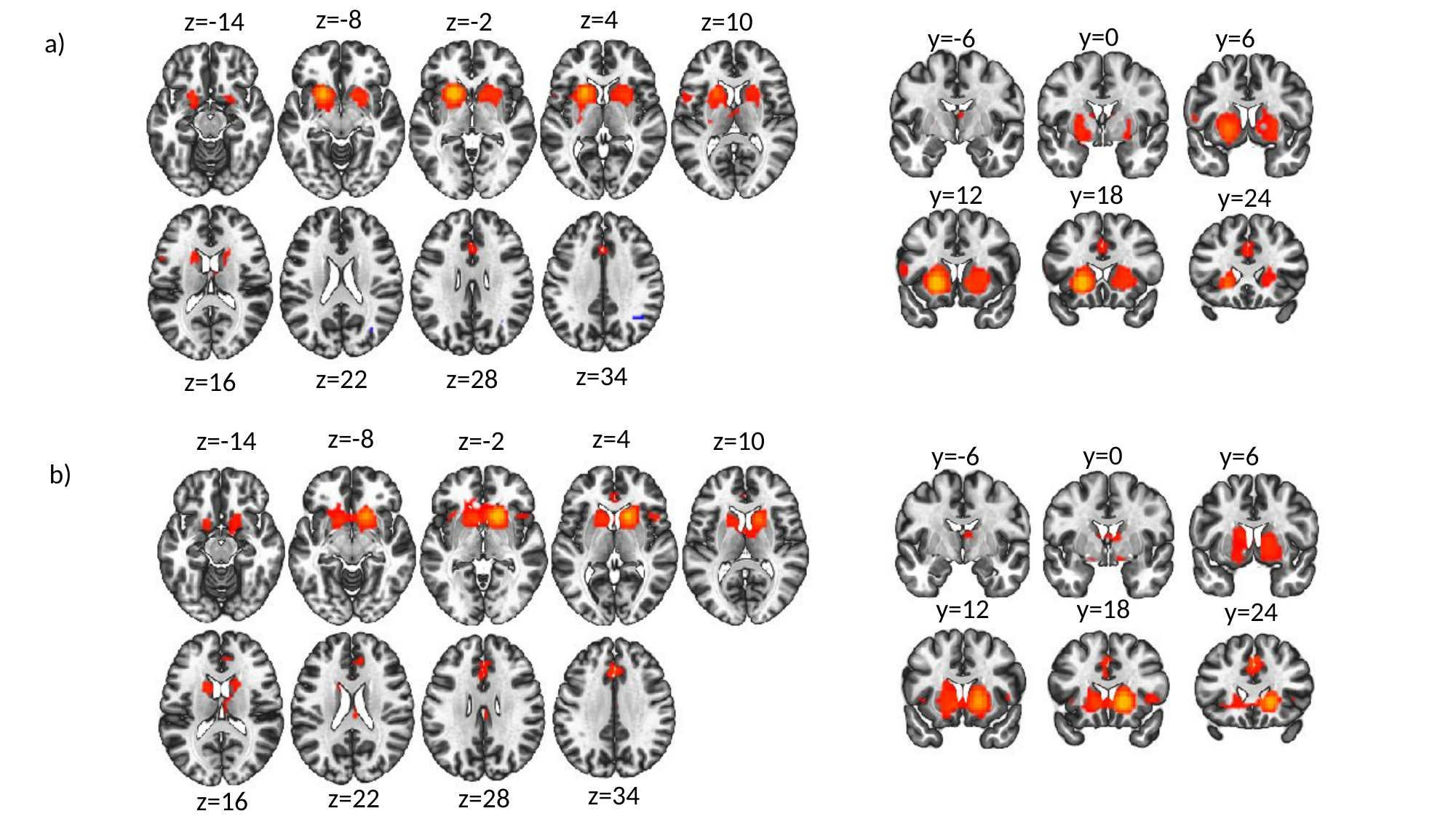

z=-8
z=4
z=-14
z=-2
z=10
y=0
y=-6
y=6
a)
y=12
y=18
y=24
z=34
z=22
z=28
z=16
z=-8
z=4
z=-14
z=-2
z=10
y=0
y=-6
y=6
b)
y=12
y=18
y=24
z=34
z=22
z=28
z=16

Supplement: S6 Fig — Seed-to-voxel functional connectivity maps showing the strength of the correlation between seeds in the left (a) and right (b) ventral striatum identified in the correlation with DV in the charity condition (x, y, z = : -21, 14, −2; x, y, z = 15, 17, −2), using the CONN toolbox [94]. Cluster FDR-corrected p < 0.001. Positive correlations are shown in red and negative in blue. DV, decision value; FDR, false discovery rate. (PPTX) [file pbio.3000283.s007.pptx]
