# Supplementary material for: Neurocomputational mechanisms at play when weighing concerns for extrinsic rewards, moral values, and social image
Source: PLoS Biol. 2019 Jun 6;17(6):e3000283. doi: 10.1371/journal.pbio.3000283 (PMC6553686; doi:10.1371/journal.pbio.3000283)
Supplement: S1 Table — AIC,; BIC,. (DOCX) [file pbio.3000283.s008.docx]

**Table S1. Comparisons of the model fit (AIC and BIC) of probit and logistic models of model 1 (first 4 lines) and model 4 (lines 5 to 8).**
