# Supplementary material for: Neurocomputational mechanisms at play when weighing concerns for extrinsic rewards, moral values, and social image
Source: PLoS Biol. 2019 Jun 6;17(6):e3000283. doi: 10.1371/journal.pbio.3000283 (PMC6553686; doi:10.1371/journal.pbio.3000283)
Supplement: S2 Table — Results of the random effect logistic regression analyses for the behavioral models 1 to 4. (DOCX) [file pbio.3000283.s009.docx]

**Table S2 (related to behavioral results and Figs. 2, 3 and 4): Results of the random effect logistic regression analyses for the behavioral models 1 to 4.**
