# Supplementary material for: Neurocomputational mechanisms at play when weighing concerns for extrinsic rewards, moral values, and social image
Source: PLoS Biol. 2019 Jun 6;17(6):e3000283. doi: 10.1371/journal.pbio.3000283 (PMC6553686; doi:10.1371/journal.pbio.3000283)
Supplement: S3 Table — Brain areas whose activity significantly correlated with DV for the negatively and the positively evaluated organizations (MNI coordinates and statistic t). DV, decision value; MNI, Montreal Neurological Institute. (DOCX) [file pbio.3000283.s010.docx]

| **Table S3 (related to Fig 3A and Fig 4A): Brain areas whose activity significantly correlated with Decision value for the negatively and the positively valued organizations (MNI coordinates and statistic t).** | | | | | | | | |
| --- | --- | --- | --- | --- | --- | --- | --- | --- |
| **Regions** | **Laterality** | **Nb. of voxels** |  | **x** | **y** | **z** |  | **t** |
|  |  |  |  |  |  |  |  |  |
| **a. Negatively valued organization: activity modulated by DV (positive correlation)** | |  |  |  |  |  |  |  |
| Anterior insula* | L | 13 |  | -36 | 14 | 1 |  | 3.88 |
| Anterior insula* | R | 10 |  | 36 | 26 | -5 |  | 3.86 |
| Middle frontal gyrus* | L | 9 |  | -48 | 44 | 16 |  | 3.57 |
|  |  |  |  |  |  |  |  |  |
| **b. Positively valued organization: activity**  **modulated by DV (negative correlation)** | |  |  |  |  |  |  |  |
| Ventral putamen* | L | 27 |  | -21 | 14 | -5 |  | 3.97 |
| * p<0,05 FWE corrected in SVC; DV: Decision Value. | | | | | | | | |
