# Supplementary material for: Neurocomputational mechanisms at play when weighing concerns for extrinsic rewards, moral values, and social image
Source: PLoS Biol. 2019 Jun 6;17(6):e3000283. doi: 10.1371/journal.pbio.3000283 (PMC6553686; doi:10.1371/journal.pbio.3000283)
Supplement: S4 Table — In the positively evaluated organization, brain regions engaged with selection of the prosocial option, and brain regions showing increasing activity with higher moral benefit. (a) Brain region whose activity was engaged with selection of the prosocial option (accept > reject) for the charity. (b) Brain region whose activity increased with higher moral benefit for accepted trials only in the charity condition (MNI coordinates and statistic t). MNI, Montreal Neurological Institute. (DOCX) [file pbio.3000283.s011.docx]

| **Table S4 (related to Fig 4B): (a) Brain region whose activity was engaged with selection of the pro-social option (accept>reject) for the charity; (b) Brain region whose activity increased with higher moral benefit for accepted trials only in the charity condition (MNI coordinates and statistic t).** | | | | | | | | |
| --- | --- | --- | --- | --- | --- | --- | --- | --- |
| **Regions** | **Laterality** | **Nb. of voxels** |  | **x** | **y** | **z** |  | **t** |
|  |  |  |  |  |  |  |  |  |
| **a. Charity: accept > reject** |  |  |  |  |  |  |  |  |
| Ventromedial prefrontal cortex (BA 11)* | R | 18 |  | 9 | 50 | -11 |  | 4.68 |
|  |  |  |  |  |  |  |  |  |
| **b. Charity: positive correlation with moral benefit for accepted trials** | | | | | | | | |
| Ventromedial prefrontal cortex (BA 11)* | R | 11 |  | 12 | 56 | -5 |  | 4.47 |
| **c. Charity: positive correlation with subject's payoff for accepted trials** | | | | | | | | |
| No cluster survived multiple comparison correction | |  |  |  |  |  |  |  |
|  |  |  |  |  |  |  |  |  |
| p < 0.001 voxel-wise uncorrected and p < 0.05 FWE cluster-wise; * p<0,05 FWE corrected in SVC; BA: Brodmann area. | | | | | | | | |
