# Supplementary material for: Neurocomputational mechanisms at play when weighing concerns for extrinsic rewards, moral values, and social image
Source: PLoS Biol. 2019 Jun 6;17(6):e3000283. doi: 10.1371/journal.pbio.3000283 (PMC6553686; doi:10.1371/journal.pbio.3000283)
Supplement: S5 Table — MNI coordinates and statistic t for the main effect of audience (public > private) and the main effect of privacy (private > public), regardless of choices. (DOCX) [file pbio.3000283.s012.docx]

| **Table S5. (related to Fig 5): MNI coordinates and statistic t for the main effect of audience (public > private) and the main effect of privacy (private > public), regardless of choices.** | | | | | | | | |
| --- | --- | --- | --- | --- | --- | --- | --- | --- |
| Regions | Laterality | Nb. of voxels |  | x | y | z |  | t |
|  |  |  |  |  |  |  |  |  |
| **a. public > private** |  |  |  |  |  |  |  |  |
| anterior cingulate cortex | R | 364 |  | 6 | 8 | 37 |  | 6.11 |
| posterior superior temporal gyrus (BA41) | R | 195 |  | 48 | -40 | 13 |  | 5.15 |
| Anterior insula* | R | 55 |  | 45 | 5 | 7 |  | 5.04 |
| Anterior insula / rolandic operculum | L | 345 |  | -45 | -4 | 13 |  | 4.69 |
|  |  |  |  |  |  |  |  |  |
| **b. private > public** |  |  |  |  |  |  |  |  |
| cerebellum | R | 329 |  | 30 | -82 | -20 |  | 5.33 |
| inferior parietal lobule (BA40) | L | 94 |  | -54 | -49 | 52 |  | 4.86 |
| inferior parietal lobule / Angular gyrus (BA38) | R | 159 |  | 51 | -64 | 46 |  | 4.46 |
| occipital gyrus | R | 366 |  | 0 | -88 | -8 |  | 4.51 |
| middle frontal gyrus | R | 44 |  | 18 | 44 | 49 |  | 4.05 |
| p < 0.001 voxel-wise uncorrected and p < 0.05 FWE cluster-wise, except in ROI (indicated by *) in which SVC was used with p<0.05 FWE voxel-wise. | | | | | | | | |
