# Supplementary material for: Neurocomputational mechanisms at play when weighing concerns for extrinsic rewards, moral values, and social image
Source: PLoS Biol. 2019 Jun 6;17(6):e3000283. doi: 10.1371/journal.pbio.3000283 (PMC6553686; doi:10.1371/journal.pbio.3000283)
Supplement: S6 Table — Brain regions showing correlations with single attributes, i.e., monetary cost, monetary benefit, moral cost, and moral benefit (MNI coordinates and statistic t). MNI, Montreal Neurological Institute. (DOCX) [file pbio.3000283.s013.docx]

| **Table S6 (related to Fig S3 and S4): Brain regions showing correlations with single attributes, i.e monetary cost, monetary benefit and moral cost, moral benefit (MNI coordinates and statistic t).** | | | | | | | | |
| --- | --- | --- | --- | --- | --- | --- | --- | --- |
| Regions | Laterality | Nb. of voxels |  | x | y | z |  | t |
|  |  |  |  |  |  |  |  |  |
| **a. NEG ORG : negative correlation with moral cost** | | | | |  |  |  |  |
| Intra-parietal region | R | 155 |  | 42 | -43 | 52 |  | 4.71 |
| Lingual gyrus | L | 72 |  | -15 | -82 | 7 |  | 4.65 |
| Middle frontal gyrus | R | 86 |  | 48 | 44 | 16 |  | 4.47 |
|  | R | 64 |  | 36 | 2 | 58 |  | 3.94 |
| Anterior insula* | R | 15 |  | 30 | 23 | -8 |  | 4.1 |
|  |  |  |  |  |  |  |  |  |
| **b. NEG ORG : positive correlation with monetary benefit** | | | | |  |  |  |  |
| Occipital lobe | L | 264 |  | -27 | -100 | 4 |  | 7.45 |
| Occipital lobe | R | 79 |  | 18 | -76 | 7 |  | 4.53 |
| Anterior insula* | L | 36 |  | -33 | 20 | 4 |  | 4.6 |
| Anterior insula* | R | 13 |  | 33 | 23 | -5 |  | 3.99 |
| Inferior frontal gyrus | R | 63 |  | 60 | 14 | 28 |  | 4.54 |
| Intra parietal cx | R | 81 |  | 45 | -46 | 52 |  | 4.08 |
|  |  |  |  |  |  |  |  |  |
| **c. POS ORG : positive correlation with moral benefit** | | | | |  |  |  |  |
| Ventromedial prefrontal cortex (BA11)* | R | 39 |  | 12 | 50 | -5 |  | 4.37 |
|  |  |  |  |  |  |  |  |  |
| **d. POS ORG : negative correlation with monetary cost** | | | | |  |  |  |  |
| No cluster survived correction for multiple comparison | | | | | | | | |
| p < 0.001 voxel-wise uncorrected and p < 0.05 FWE cluster-wise, except in ROI (indicated by *) in which SVC was used with p<0.05 FWE. POS ORG: positively evaluated organization; NEG ORG: negatively evaluated organization; BA: Brodmann area | | | | | | | | |
