# Supplementary material for: Neurocomputational mechanisms at play when weighing concerns for extrinsic rewards, moral values, and social image
Source: PLoS Biol. 2019 Jun 6;17(6):e3000283. doi: 10.1371/journal.pbio.3000283 (PMC6553686; doi:10.1371/journal.pbio.3000283)
Supplement: S7 Table — Functionally interconnected brain regions (seed to voxel) with DV-related anterior insula in the negatively evaluated organization. Seed anterior insula Left ROI (a) is a 4 mm radius sphere with coordinates x, y, z = −36, 14, −1, and seed anterior insula Right ROI is a 4 mm sphere with coordinates x, y, z = 36, 20, 1. MNI coordinates of peak. DV, decision value; MNI, Montreal Neurological Institute; ROI, region of interest. (DOCX) [file pbio.3000283.s014.docx]

| **Table S7 (related to Fig S5): Functionally interconnected brain regions (seed to voxel) with decision value-related anterior insula in the negatively valued organization. Seed anterior insula Left ROI (a) is a 4mm radius sphere with coordinates x,y,z= -36,14,-1, and Seed anterior insula Right ROI is a 4mm sphere with coordinates x,y,z=36,20,1. MNI coordinates of peak.** | | | | | | | | | |
| --- | --- | --- | --- | --- | --- | --- | --- | --- | --- |
| Regions | Laterality | Nb. of voxels |  | x | y | z |  | |  |
|  |  |  |  |  |  |  |  | |  |
| **a. Seed Left Anterior insula** |  |  |  |  |  |  |  | |  |
| Anterior insula | L | 4446 |  | -36 | 14 | 00 | |  |  |
| Anterior Cingulate Gyrus | R | 2825 |  | 00 | 20 | 36 | |  |  |
| Anterior insula Cortex | R | 2821 |  | 46 | 16 | -6 | |  |  |
| Supramarginal Gyrus Right | R | 398 |  | 60 | -38 | 36 | |  |  |
| Supramarginal Gyrus Left | L | 223 |  | -54 | -38 | 30 | |  |  |
| Frontal Pole Right | R | 153 |  | 34 | 50 | 30 | |  |  |
|  |  |  |  |  |  |  | |  |  |
| **b. Seed Right anterior insula** |  |  |  |  |  |  | |  |  |
| Anterior insula | R | 6802 |  | 36 | 20 | 00 | |  |  |
| Paracingulate Gyrus Right | R | 1897 |  | 12 | 28 | 34 | |  |  |
| Anterior insular | L | 1552 |  | -36 | 14 | 04 | |  |  |
| Anterior Supramarginal Gyrus Right | R | 721 |  | 58 | -38 | 52 | |  |  |
| Frontal Pole Left | L | 507 |  | -36 | 44 | 16 | |  |  |
|  | R | 186 |  | 10 | -22 | 00 | |  |  |
| Functional connectivity analysis is thresholded with a voxel-level p-FDR-corrected < 0.005 and a cluster-level p-FDR-corrected < 0.001 | | | | | | | | | |
