# Supplementary material for: Neurocomputational mechanisms at play when weighing concerns for extrinsic rewards, moral values, and social image
Source: PLoS Biol. 2019 Jun 6;17(6):e3000283. doi: 10.1371/journal.pbio.3000283 (PMC6553686; doi:10.1371/journal.pbio.3000283)
Supplement: S8 Table — Functionally interconnected brain regions (seed to voxel) with the DV-related ventral putamen in the positively evaluated organization. Seed ventral putamen Left ROI (a) is a 4 mm radius sphere with coordinates x, y, z = −21, 14, −2, and seed ventral putamen Right ROI is a 4 mm sphere with coordinates x, y, z = 15, 17, −2. MNI coordinates of peak. DV, decision value; MNI, Montreal Neurological Institute; ROI, region of interest. (DOCX) [file pbio.3000283.s015.docx]

| \| **Table S8 (related to Fig S6): Functionally interconnected brain regions (seed to voxel) with the decision value-related ventral putamen in the positively valued organization. Seed ventral putamen Left ROI (a) is a 4mm radius sphere with coordinates x,y,z=-21,14,-2, and Seed ventral putamen Right ROI is a 4mm sphere with coordinates x,y,z=15,17,-2. MNI coordinates of peak.** \| \| \| \| \| \| \| \| \| \| \| --- \| --- \| --- \| --- \| --- \| --- \| --- \| --- \| --- \| --- \| \| Regions \| Laterality \| Nb. of voxels \|  \| x \| y \| z \|  \| \| \|  \|  \|  \|  \|  \|  \|  \|  \| \| \| **a. Seed Left Ventral putamen** \|  \|  \|  \|  \|  \|  \|  \| \| \| Putamen Left \| L \| 1762 \|  \| -20 \| 14 \| -02 \| \| \| Accumbens Right \| R \| 1040 \|  \| 12 \| 10 \| -06 \| \| \| Anterior Cingulate Gyrus \| R \| 86 \|  \| 00 \| 20 \| 30 \| \| \|  \|  \|  \|  \|  \|  \|  \| \| \|  \|  \|  \|  \|  \|  \|  \| \| \| **b. Seed Right ventral putamen** \|  \|  \|  \|  \|  \|  \| \| \| Putamen Right \| R \| 2243 \|  \| 16 \| 16 \| -02 \| \| \| Anterior Cingulate Gyrus \| R \| 294 \|  \| 00 \| 22 \| 40 \| \| \|  \| \| \| \| \| \| \| \| \| \| |
| --- | --- | --- | --- | --- | --- | --- | --- | --- | --- | --- | --- | --- | --- | --- | --- | --- | --- | --- | --- | --- | --- | --- | --- | --- | --- | --- | --- | --- | --- | --- | --- | --- | --- | --- | --- | --- | --- | --- | --- | --- | --- | --- | --- | --- | --- | --- | --- | --- | --- | --- | --- | --- | --- | --- | --- | --- | --- | --- | --- | --- | --- | --- | --- | --- | --- | --- | --- | --- | --- | --- | --- | --- | --- | --- | --- | --- | --- | --- | --- | --- | --- | --- | --- | --- | --- | --- | --- | --- | --- | --- | --- | --- | --- | --- | --- | --- | --- | --- | --- | --- | --- | --- | --- | --- | --- | --- | --- | --- | --- | --- | --- |

Functional connectivity analysis is thresholded with a voxel-level p-FDR-corrected < 0.005 and a cluster-level p-FDR-corrected < 0.001
